# Genetic determinants in the development of sensitization to environmental allergens in early childhood

**DOI:** 10.1002/iid3.38

**Published:** 2014-11-20

**Authors:** Priya Tripathi, Xiumei Hong, Deanna Caruso, Peisong Gao, Xiaobin Wang

**Affiliations:** 1 Division of Allergy and Clinical Immunology, Johns Hopkins University School of MedicineBaltimore, Maryland, 21224; 2 Center on Early Life Origins of Disease, Department of Population, Family and Reproductive Health, Johns Hopkins University Bloomberg School of Public HealthBaltimore, Maryland, 21205

**Keywords:** Asthma, allergic sensitization, cockroach sensitization, house dust mites, single nucleotide polymorphisms

## Abstract

Sensitization to environmental allergens remains one of the strongest risk factors for asthma, and there is likely a genetic basis. We sought to identify genetic determinants for the development of allergic sensitization to environmental allergens, particularly cockroach allergen, in early childhood. A total of 631 children with the information about genotypic data on 895 single nucleotide polymorphisms (SNPs) in 179 candidate genes were selected from an existing dataset (Boston Birth Cohort). Genetic analysis was performed for allergic sensitizations among all subjects and sub-population, Black/African, respectively. Eight SNPs in seven genes showed significant association with allergic sensitization with *P* < 0.05, including two top SNPs, rs7851969 in *JAK2 (P* *=* 0.003*)* and rs11739089 in *CNOT6 (P* *=* 0.008*)*. When analyses were specifically performed for cockroach sensitization, 16 SNPs in 13 genes showed *P* < 0.05, including five genes with SNPs at *P* < 0.01 (*JAK1*, *JAK3*, *IL5RA*, *FCER1A*, and *ADAM33)*. Particularly, haplotype analyses demonstrated that multiple-haplotypes in *FCER1A* were significantly associated with cockroach sensitization with the strongest association for a 2-marker haplotype (rs6665683T-rs12136904T, *P* *=* 0.001). Furthermore, SNP rs6665683 was marginally associated with the levels of cockroach allergen specific IgE. When a similar analysis was performed for house dust mite, four SNPs in three genes (*JAK2, MAML1*, and *NOD1*) had *P* < 0.01. Of these, *JAK2* appeared to be an only gene showing association across the sensitizations we analyzed. Some of findings were further validated when analysis was limited to black population. Our study identified several loci that may confer the susceptibility to allergic sensitization, and suggested that sensitization to allergens may depend on their unique loci.

## Introduction

Asthma is the leading serious chronic illness of children in the United States [1]. It is largely accepted that gene–environment interactions are responsible for the development of asthma; cockroach sensitization has been demonstrated to be one of the major risk factors for the development of asthma [2–4]. In particular, it has been suggested that cockroach sensitized exposure may be closely associated with increased asthma morbidity among inner-city residents [5–7]. While there appears to be a rather clear relationship between allergen exposure and allergen sensitization, a dose–response relationship is mostly relevant for “susceptible” individuals [8]. Similarly, although no difference was observed in cockroach sensitization between those with and without asthma, environmental allergen exposure was suggested to play a critical role in the development of asthma among genetically susceptible individuals [9]. Furthermore, although many individuals were exposed to very high concentrations of allergen, they never become sensitized [10]. These findings suggest that cockroach sensitization is not a function of cockroach allergen exposure alone, and genetic susceptibility may be important. Indeed, Hopp et al. [11] reported that skin test reactivity is determined by genetic and environmental factors with a greater genetic influence as reflected in the higher correlation in monozygotic twins. Recent studies have demonstrated a significant familial aggregation of allergic sensitization to cockroach allergen in the Chinese population [12] and suggested a role of genetic factors in allergic sensitization. Previously Togias et al. [13] demonstrated that individuals sensitized to cockroach allergens, especially African Americans, have more severe symptoms than the general asthma population, exhibiting higher IgE levels, a high degree of steroid dependency and greater chronicity of disease. Collectively, these studies suggest that there is a genetic basis for environmental allergen sensitization, which contributes to the risk of asthma and/or the severity of asthma.

Although it is clear that there is a genetic basis for allergen induced sensitization, few studies have been performed to identify genetic factors specifically for cockroach sensitization. In a study on *HLA-D* associations and cockroach sensitization, Donfack et al. [14] observed associations with alleles of the *HLA-DR* molecule, DRB1*0101 in Hutterites and DRB1*0102 in African Americans, and hypothesized that the DRB1*0102 allele may have a higher affinity for cockroach allergens and therefore may elicit a stronger response to bind antigens than DRB1*0101 allele. In a genome-wide linkage study of asthma-related phenotypes on 2551 individuals from 533 families, Xu et al. [15] provided suggestive evidence of linkage at a possible QTL, D4S1647 for skin reactivity to cockroach defined by skin prick tests (SPT) (pointwise *P* *=* 0.0003). Several similar epidemiological studies concerning the association between genetic and SPT reactivity have found several SPT-related gene [16,17]. Furthermore, Hunninghake et al. [18] reported that there was significant evidence of linkage to IgE to cockroach on chromosome 5q23 (peak LOD, 4.14 at 127 cM) in female subjects. Within this genomic region, there is a compelling candidate gene with experimental evidence of female-specific effects on lung disease, thymic stromal lymphopoietin (TSLP). In a sex-stratified analysis, the single-nucleotide polymorphism (SNP) rs2289276, in the 5′untranslated region of TSLP was associated with reductions in specific IgE to cockroach in Costa Rican girls. In addition, Leung et al. [19] observed that polymorphisms in the Mannose-binding lectin (MBL) gene, a member of the innate immune system, may protect against cockroach sensitization in Chinese children, and Pistiner et al. [20] demonstrated that polymorphisms in IL-12A were associated with cockroach sensitization among children with asthma in both Costa Rica and Caucasian children with asthma in the Childhood Asthma Management Program (CAMP). In addition, new information is emerging from hypothesis-free approaches such as genome-wide association studies. Recent study by Bønnelykke et al. [21] have identified ten loci influencing allergic sensitization by meta-analysis of genome-wide association studies, including SNPs in or near *TLR6*, *C11orf30*, *STAT6*, *SLC25A46*, *HLA-DQB1*, *IL1RL1*, *LPP*, *MYC*, *IL2*, and *HLA-B* on adult population. Interestingly, some of those identified genes have been shown to be critical in the pathogenesis of allergic diseases.

In this study, we examined the genetic determinants for the development of sensitization to specific environmental allergens in early childhood in a total of 631 Children consisting of allergen sensitized and non-sensitized individuals, all of whom were identified from Boston Birth Cohort (BBC) and have the genotyping data of 895 SNPs in 179 highly selected genes. Our initial analysis was performed for allergic sensitization to common environmental allergens, followed by an analysis specific to cockroach allergen and house dust mite. Findings from these initial analyses were further validated when analyses were limited to black population only. This study provided target genes for future replication in diverse ethnic populations and further functional investigation.

## Materials and Methods

### Study population

**Figure 1:**
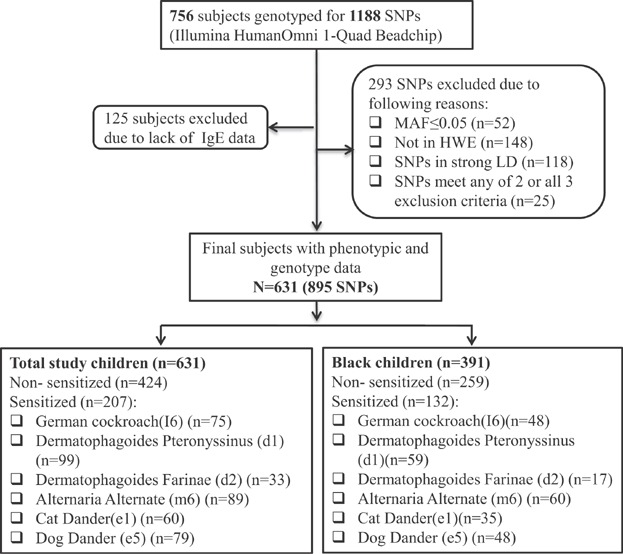
Flow chart on study sample selection from the Boston Birth Cohort.

A total of 1219 children from the BBC were initially genotyped for 1188 SNPs. Detailed information on the recruitment of BBC has been described previously [22]. In this study, we specifically focused on subjects who have both genotypic data and data on allergic sensitization. As shown in Figure 1, there are a total of 756 children from BBC with 1188 SNPs genotyped by the Illumina HumanOmni-Quad Beadchip. Of these, 125 subjects were further removed because of failure in quality control (QC) measures. In addition, we removed 293 SNPs because of one or more of the followings: (1) minor allele frequency (MAF) ≤5% (n *=* 52), (2) Hardy–Weinberg equilibrium (HWE) *P*-value <0.001 (n *=* 148), (3) SNPs in strong linkage disequilibrium (LD—r2 ≥ 0.8; n *=* 118), and (4) SNPs with 2 or more than 2 mentioned criteria above (n *=* 25) [23]. Thus, our final analysis was performed in 631 subjects consisting of allergen sensitized (AS, n *=* 207) and non-sensitized to environmental allergens (n *=* 424; Table1) with the information about genotypic data on 895 single nucleotide polymorphisms (SNPs) in 179 candidate genes (Table S1). Allergic sensitization was defined as at least 1 positive allergen-specific IgE measurement. These allergic individuals incldued those who are sensitized to: (1) cockroach allergen (n *=* 75), (2) dermatophagoides Pteronyssinus (n *=* 99), Dermatophagoides Farinae (N *=* 33), (3) alternaria alternata (n *=* 89), (4) danger to dog (n *=* 79), and (6) cat (n *=* 60) (Fig. 1). For the black population, there were 132 allergic sensitized and 259 were non-sensitized subjects. Among these subjects with allergic sensitization, a total of 48 subjects were sensitized to cockroach, 59 were sensitized to house dust mite, and 160 were sensitized to others (Table1, Table S2). Among those subjects, the most common races are Black (62.1%), Hispanic (21.7%), and White (5.1%), Cap Verdean (5.1%), respectively. The rest are less than 5% (Asian/Pacific Islander, Caribbean, and unknown, Table2). The study protocols were approved by the Institutional Review Boards (IRBs) of Children's Memorial Hospital in Chicago and Boston University Medical Center. A data repository protocol of the BBC was approved by the Johns Hopkins Bloomberg School of Public Health IRB. All participating families provided written informed consent.

**Table 1:** Characteristics of study children (N *=* 631)

Subjects	Total	Total study children		*P*-value	Total	Black children		*P*-value
		Non-sensitized (424)	Sensitized (207)			Non-sensitized (259)	Allergen-sensitized (132)	
Age (M ± SD)	2.5 ± 2.07	2.17 ± 1.88	3.21 ± 2.27	<0.001	2.6 ± 2.08	2.29 ± 1.91	3.26 ± 2.25	0.006
Gender (F) N (%)	313 (49.6)	205 (48.3)	108 (52.2)	0.397	197 (50.4)	130 (50.2)	67 (50.8)	1.000
Maternal continuous smoking N (%)	55 (8.7)	42 (9.9)	13 (6.3)	0.136	35 (9.0)	27 (10.4)	8 (6.1)	0.191
Maternal atopy N (%)	232 (36.8)	155 (36.6)	77 (37.2)	0.930	142 (36.3)	96 (37.1)	46 (34.8)	0.775
Postnatal IgE (median)	1.37	1.17	1.87	<0.001	1.43	1.17	1.90	<0.001

**Table 2:** Sensitization to all allergens, cockroach, and house dust mite in different races

	Total	Non-sensitized	Sensitized		
			All allergens	Cockroach	House dust mite
Black/African American	391	259 (66.2%)	132 (33.8%)	48 (36.4%)	59 (44.7%)
White	32	24 (75.0%)	8 (25.0%)	1 (12.5%)	4 (50.0%)
Hispanic	137	88 (64.2%)	49 (35.8%)	23 (46.9%)	32 (65.3%)
Asian/Pacific	9	9 (100.0%)	0 (0.00)	0 (0.00)	0 (0.00)
Cap Verdean	32	22 (68.8%)	10 (31.2%)	1 (10.0%)	3 (30.0%)
Mixed	2	1 (50.0%)	1 (50.0%)	0 (0.00)	0 (0.00)
Caribbean	5	5 (100.0%)	0 (0.00)	0 (0.00)	0 (0.00)
Unknown	23	16 (72.7%)	7 (27.3%)	2 (28.6%)	1 (14.3%)
Total	631	424 (67.3%)	207 (32.7%)	75 (36.2%)	99 (47.8%)

### Measurement of specific IgE

Venous blood samples were collected and stored at −80°C for IgE measurement. Tests for serum allergen specific IgE were performed by using ImmnunoCAP Total Low Range Assay (Phadia AB, Uppsala, Sweden) according to the manufacturer's prescribed protocol for the following six common indoor extract allergens characterized by company: *German cockroach (I6); dermatophagoides Pteronyssinus (d1); dermatophagoides Farinae (d2); alternaria alternata (m6); cat dander (e1);* and *dog dander (e5)*. The calibration range for tIgE and sIgE was 2.0–5000 kU/L and 0.1–100 kU_A_/L, respectively. These assays were measured by the Clinical Immunology Laboratory at Children's Memorial Hospital, Chicago, Illinois.

### Rationale of SNP selection

We have provided a list of selected 895 SNPs with the information about allele, chromosome, chromosome position, and possible function predicted by Regulome DB score (http://regulome.stanford.edu/) in supplementary materials (Table S1). In addition to tagging SNPs with LD cut-off as r2 ≥ 0.8 [23], we selected potentially functional SNPs with a minor allele frequency of 0.05 or greater that met the following criteria: (1) coding SNPs, (2) SNPs creating/disrupting a splicing site, or (3) SNPs predicted to be functional variants based on bioinformatics tools (e.g., PupaSuite [http://pupasuite.bioinfo.cipf.es/], FuncPred [http://snpinfo.niehs.nih.gov/snpfunc.htm], and F-SNP [http://compbio.cs.queensu.ca/F-SNP/]; for example, this included SNPs in transcription factor binding sites, in exonic splicing enhancers or silencers, in microRNAs and their target sequences, and/or in a DNA triplex [triplex]); and (4) SNPs within the expression quantitative trait locus. Additional clinical and genetic data are reported with details in our previous published articles [23–28]. Furthermore, for candidate gene selection, we selected those that may be associated with asthma, eczema and other allergic diseases, and genes that are located in functionally important pathways such as antigen presentation, IgE production, innate immunity, Th1 and Th2 skewing, and airway remodeling.

### SNP genotyping

SNPs were genotyped in the genotyping center at Washington University at St Louis by using the Illumina GoldenGate custom panel (Illumina, Inc., San Diego, CA). For quality control and quality assurance, four duplicate DNA control samples were included in each 96-well plate and were genotyped. The concordance rate of these duplicate samples was greater than 99.5%.

### Statistical analysis

To control for potential confounding caused by population stratification, 150 ancestry informative markers, with averaged δ (allele frequency difference between two ancestral populations) ≥0.5, were randomly selected from a recently reported genome-wide admixture map [29]. Of those, 144 ancestry informative markers (with a call rate ≥98.0%) were included in the estimation of ancestral proportion for three ancestral populations (Asian, European, and African) using the STRUCTURE program (version 2.3.1; http://pritch.bsd.uchicago.edu/structure.html). These estimated ancestral proportion was then included as a covariate in subsequent analyses. Detailed information can be found previously published paper [23].

For each SNP genotyped in this study, a χ^2^ test was applied to test HWE in the total population. Pairwise LD of SNPs in each gene was also calculated. Association between each individual SNP and allergic sensitization was performed using logistic regression analysis under an additive model, with adjustments for age, gender and race. The levels of total and specific IgE were log-transformed from a non-normal distribution into a normal distribution. The association with IgE was assessed using a linear regression analysis adjusted for age, gender and race. Similar analyses were performed for the black population adjusted for age and gender. Expectation–maximization algorithm was used to analyze haplotype frequencies and *P* values. To represent final results omnibus *P* values were recorded. Omnibus test is a general name refers to an overall or a global test and in commonly omnibus test is called in other expressions such as: *F*-test or Chi-squared test. Omnibus test as a statistical test is implemented on an overall hypothesis that tends to find general significance between parameters' variance. All data analyses were conducted by using the PLINK program (http://pngu.mgh.harvard.edu/∼purcell/plink/). A 2-tailed *P* value of less than 0.05 was considered statistically significant.

## Results

### Characteristics of study population

Total study population consisted of approximately equal proportion of male subjects between allergens sensitized (52.2%) and non-sensitized (48.3%; *P* *=* 0.397; Table1) and equal proportion of maternal atopy between allergen sensitized (37.2%) and non- sensitized (36.6%; *P* *=* 0.93), and equal proportion of maternal continuous smoking between allergen sensitized (6.3%) and non- sensitized (9.9%; *P* *=* 0.136). However, there were significant differences between two groups for age (allergens sensitized vs non-sensitized to environmental allergens, 3.21 ± 2.27 vs. 2.17 ± 1.88 (years), *P* < 0.001). Total serum IgE levels (KU/L) were significantly higher among those who are sensitized to one of the tested allergens as compared to those who are not sensitized to any of these allergens (median of log-transformed IgE, 1.87 vs. 1.17, *P* < 0.001). When analysis was limited to Black children, the significant difference was also observed for age (allergens sensitized vs. non-sensitized to environmental allergens, 3.26 ± 2.25 vs. 2.29 ± 1.91 (years), *P* *=* 0.006), and postnatal IgE (median of log-transformed IgE, KU/L) allergens (sensitized vs. non-sensitized to environmental allergens, 1.90 vs. 1.17, *P* < 0.001). When all these allergic subjects were divided by race, there was no significant difference among these most common races (n ≥ 10) [Black (33.8%), Hispanic (35.8%), and Cap Verdean (31.2%)]. The same is also true for those who are specifically allergic to cockroach [Black (36.4%) and Hispanic (46.9%)]. However, the difference was observed for those who are allergic to house dust mite in Hispanic and Black/African (65.3% vs. 44.7%, *P* *=* 0.0027; Table2).

### Association of SNPs with allergic sensitization

**Figure 2:**
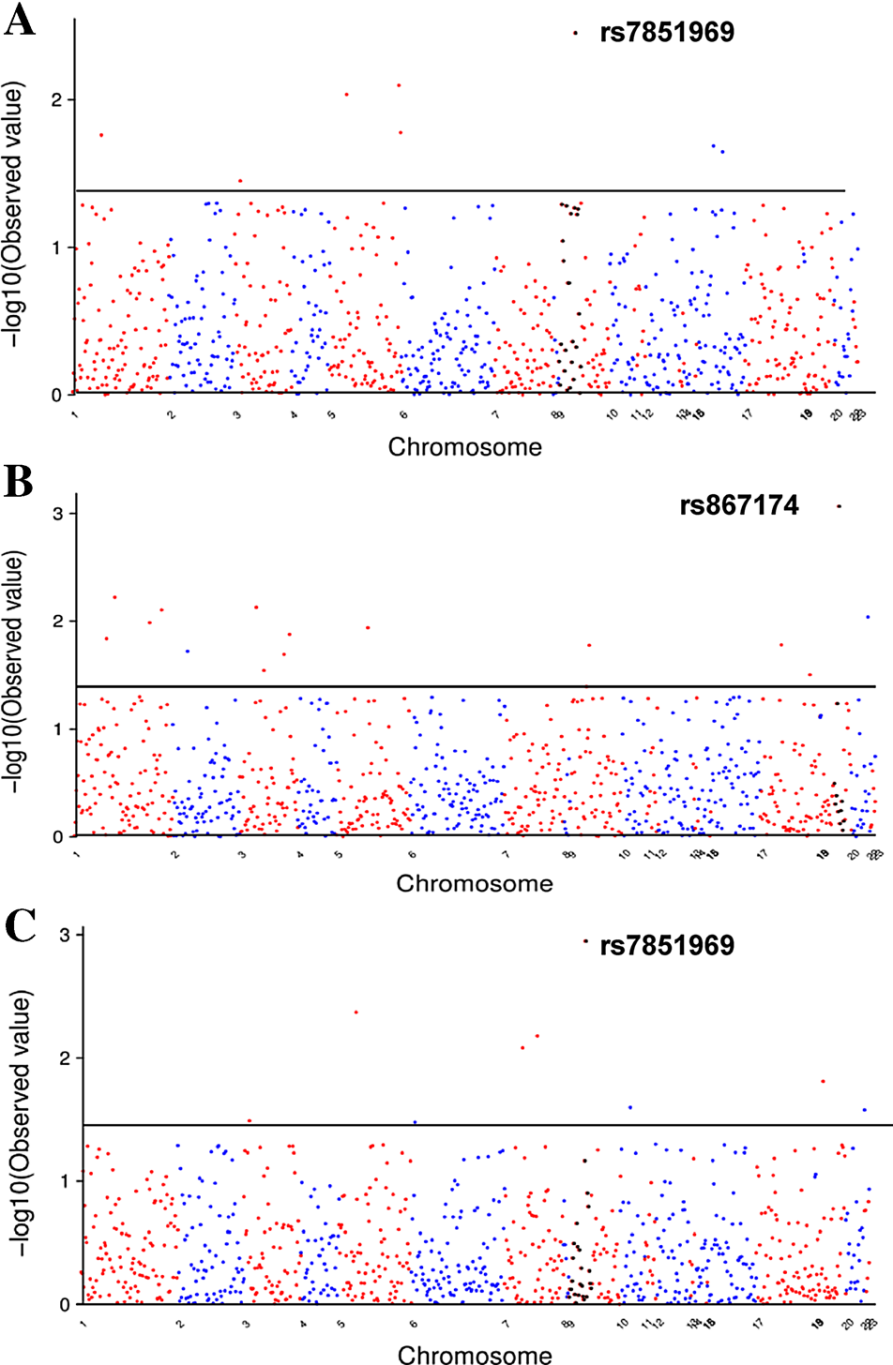
The −log_10_ (*P* value) for associations of *genotyped* SNPs with (A) allergic sensitization, (B) cockroach allergen, and (C) house dust mite. The x-axis represents individual SNP. The gray line represents *P* *=* 0.05.

We examined the association between those genotyped SNPs and allergic sensitization (Table3, Fig. 2A). A total of 8 SNPs in or near seven genes showed *P* < 0.05. Of these, three SNPs had *P* < 0.01, including rs7851969 in *JAK2* [Odds ratio (OR) (95% CI), 2.08 (1.29–3.37), *P* *=* 0.003], rs11739089 in *CNOT6* [OR (95% CI), 1.54 (1.17–2.11), *P* *=* 0.008], and rs6627 in *MAML1* [OR (95% CI), 0.70 (0.54–0.92), *P* *=* 0.009]. Furthermore, several candidate genes also showed significant association with allergic sensitization, including *IL4R* (rs3024633, rs3024576), *JAK1* (rs7524842), *IL12B* (rs3212227), and *IL5RA* (rs163550). When the same analyses was limited to black population, we found that, except for SNPs in gene *MAML1* and *IL5RA*, the associations we observed for others in total population still remained. Of these, SNP rs11739089 in *CNOT6* showed even stronger association in the black population (*P* = 0.001).

**Table 3:** Association between genotyped single gene polymorphisms and sensitization to environmental allergens

rs ID	Gene	Chromosome	SNP	Minor allele frequency total		OR (95% CI)	*P*-value	Black Non-sensitized (259) vs. sensitized (132)	
				Non-sensitized (N *=* 424)	Sensitized (N *=* 207)			OR (95% CI)	*P*-value
rs7851969	JAK2	9	C/G	0.05	0.09	2.08 (1.29–3.37)	0.003	2.02 (1.17–3.49)	0.011
rs11739089	CNOT6	5	T/C	0.13	0.18	1.54 (11.70–2.11)	0.008	1.89 (1.28–2.78)	0.001
rs6627	MAML1	5	G/A	0.38	0.31	0.70 (0.54–0.92)	0.009	0.84 (0.61–1.14)	0.835
rs3024633	IL4R	16	A/G	0.08	0.04	0.53 (0.30–0.91)	0.022	0.47 (0.24–0.92)	0.027
rs7524842	JAK1	1	T/C	0.05	0.08	1.87 (1.12–3.13)	0.017	1.81 (1.03–3.17)	0.040
rs3212227	IL12B	5	A/C	0.33	0.41	1.36 (1.06–1.75)	0.017	1.38 (1.01–1.88)	0.044
rs3024576	IL4R	16	G/A	0.10	0.07	0.57 (0.35–0.91)	0.020	0.45 (0.25–0.83)	0.010
rs163550	IL5RA	3	G/C	0.16	0.22	1.39 (1.02–1.90)	0.035	1.22 (0.80–1.85)	0.357

OR, odds ratio; CI, confidence interval; all *P* values were analyzed under additive model adjusted for age, gender and race.

### Association of SNPs with sensitization to cockroach allergen

When analysis was performed specifically for cockroach sensitization as defined by positive specific IgE measurement of German cockroach (I6), 17 SNPs in 11 genes showed *P* < 0.05 (Table4, Fig. 2B). The strongest association was observed for SNP rs867174 in *JAK3* [OR (95% CI), 0.28 (0.13–0.61), *P* *=* 0.001]. Additionally, five SNPs in four genes had *P* < 0.01, including, SNP rs10889502 in *JAK1* (*P* *=* 0.006), rs334807 in *IL5RA* (*P* *=* 0.007), both rs6665683 and rs12135788 in *FCER1A* (*P* *=* 0.008 and 0.010, respectively), and rs2787094 in ADAM33 (*P* *=* 0.009). Furthermore, several well-recognized candidate genes also showed association with allergic sensitization, including *IL13* (rs1800925, *P* *=* 0.016, rs2069743, *P* *=* 0.04), *JAK2* (rs10815160, *P* *=* 0.017), *DPP10* (rs4516432, *P* *=* 0.019), and *CD86* (rs1129055, *P* *=* 0.013; rs11717893, *P* *=* 0.020). Similarly, when analysis was restricted to black population, we found similar associations for several top SNPs with *P* < 0.01, including SNP in *JAK3* (rs867174, *P* *=* 0.002), *JAK1* (rs10889502, *P* *=* 0.011), *FCER1A* (rs6665683, *P* *=* 0.013; rs12135788, *P* *=* 0.005), and *ADAM33* (rs2787094, *P* *=* 0.036), and for few others, *IL13* (rs1800925, *P* *=* 0.016) and *CD86* (rs1129055, *P* *=* 0.011; rs11717893, *P* *=* 0.007).

**Table 4:** Association between genotyped single gene polymorphisms and cockroach sensitization

rs ID	Gene	Chromosome	SNP	Minor allele frequency total		OR (95% CI)	*P*-value	Black non-sensitized (259) vs. sensitized (48)	
				Non-sensitized (N *=* 424)	Sensitized (N *=* 75)			OR (95% CI)	*P*-value
rs867174	JAK3	19	A/G	0.16	0.05	0.28 (0.13–0.61)	0.001	0.19 (0.07–0.55)	0.002
rs10889502	JAK1	1	C/G	0.42	0.54	1.71 (1.70–2.50)	0.006	1.88 (1.16–3.07)	0.011
rs334807	IL5RA	3	G/T	0.46	0.57	1.67 (1.15–2.41)	0.007	1.45 (0.92–2.28)	0.106
rs6665683	FCER1A	1	C/T	0.15	0.25	1.81 (1.17–2.82)	0.008	1.87 (1.14–3.05)	0.013
rs2787094	ADAM33	20	C/G	0.38	0.27	0.59 (0.39–0.87)	0.009	0.59 (0.37–0.97)	0.036
rs12135788	FCER1A	1	G/T	0.24	0.35	1.63 (1.12–2.37)	0.010	1.94 (1.22–3.09)	0.005
rs1800925	IL13	5	T/C	0.31	0.43	1.61 (1.12–2.33)	0.011	1.71 (1.10–2.64)	0.016
rs1129055	CD86	3	A/G	0.21	0.12	0.51 (0.30–0.87)	0.013	0.39 (0.19–0.81)	0.011
rs17127114	JAK1	1	G/A	0.16	0.08	0.46 (0.25–0.86)	0.015	0.48 (0.22–1.07)	0.072
rs10815160	JAK2	9	G/T	0.38	0.27	0.61 (0.40–0.92)	0.017	0.61 (0.36–1.05)	0.074
rs1635278	CCL16	17	A/G	0.42	0.31	0.62 (0.42–0.92)	0.017	0.66 (0.42–1.03)	0.070
rs4516432	DPP10	2	T/C	0.17	0.24	1.75 (1.09–2.79)	0.019	1.77 (0.95–3.29)	0.073
rs11717893	CD86	3	C/T	0.23	0.32	1.59 (1.08–2.35)	0.020	1.92 (1.19–3.09)	0.007
rs11079339	EPX	17	A/G	0.13	0.2	1.71 (1.07–2.73)	0.024	2.01 (1.12–3.62)	0.020
rs4054760	IL5RA	3	T/A	0.22	0.32	1.57 (1.05–2.23)	0.029	1.48 (0.88–2.48)	0.138
rs7851969	JAK2	9	C/G	0.05	0.09	1.99 (1.03–3.87)	0.040	1.99 (1.03–3.87)	0.082

OR, odds ratio; CI, confidence interval; all *P* values were analyzed under additive model adjusted for age, gender and race.

### *FCER1A* haplotypes contribute to cockroach sensitization

**Figure 3:**
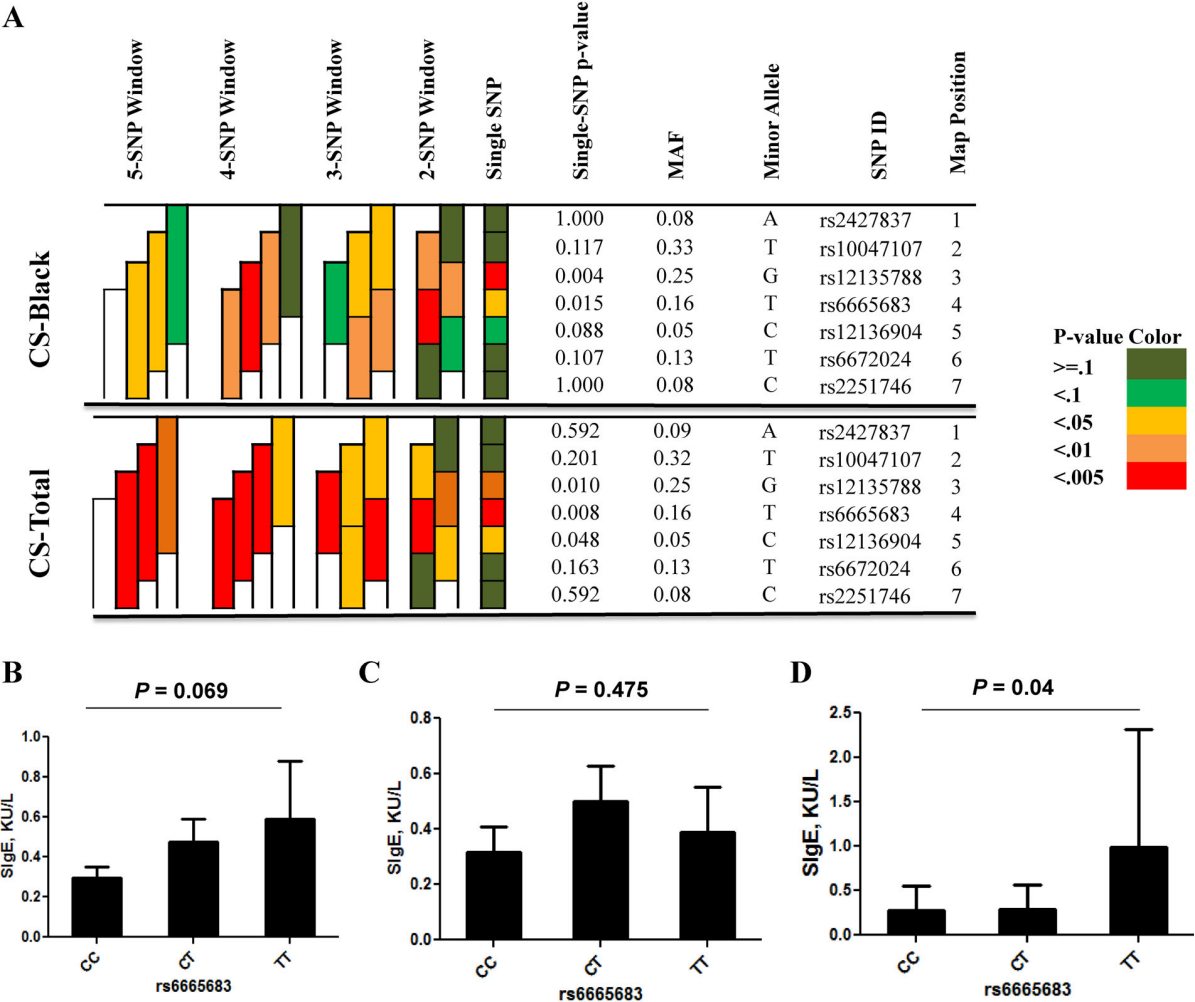
Association between *FCER1A* SNPs and cockroach sensitization. (A) Haplotype analysis for *FCER1A* SNPs and cockroach sensitization. Shading represents the magnitude and significance of SNP and 2–4 haplotype with a red to white gradient reflecting higher to lower *P* values. CS, cockroach sensitization. (B and C) Association of *FCER1A* SNP rs6665683 with sIgE was performed among all (B) or black (C) or non-black (D) subjects with cockroach sensitization.

Haplotype analyses were performed for several genes with at least two SNPs showing association with cockroach sensitization, including *JAK1, IL5RA, FCER1A, IL13*, and *CD86*. Significance was illustrated by different colors from green (*P* > 0.1) to red (*P* < 0.005). Of these, multiple haplotypes of 2–5 SNPs in *FCER1A* were significantly associated with cockroach sensitization (*P* *=* 0.042–0.001, Fig. 3A), with the strongest association seen with a 2-SNP haplotype (T-T, *P* *=* 0.001) comprised of markers in the intron region for *FCER1A* (rs6665683 and rs1213904) among all allergen sensitized subjects. The same pattern was seen when analysis was limited to black population. No association for *FCER1A* haplotypes was observed for sensitization to house dust mite among all or black population (Data not shown). The results suggest that *FCER1A* SNPs may be specific to the susceptibility to cockroach sensitization.

### *FCER1A* SNPs contribute to differential levels of cockroach allergen specific IgE

To explore the biological link of *FCER1A* SNPs to disease, we examined the association between all FCER1A SNPs and the levels of cockroach allergen specific IgE (sIgE). The analysis was performed by a linear regression analysis adjusting for age, sex, and race. The SNP rs6665683 that was associated with cockroach sensitization showed marginal association (*P* *=* 0.069, Fig. 3B). Those carrying CC genotype had much lower levels of sIgE (0.100 KU/L ± 0.096, n *=* 44) as compared to those carrying CT (0.476 KU/L ± 0.571, n *=* 25) and TT (0.589 KU/L ± 0.714, n *=* 6). While similar pattern was seen for black population, it was not statistically significant (*P* *=* 0.475, Fig. 3C). We also specifically analyzed the correlation between the genotype of FCER1A SNP rs6665683 and cockroach allergen specific IgE plasma levels in non-black subpopulation and observed that those carrying TT genotype have significantly higher levels of cockroach allergen specific IgE as compared with those with CC and CT (*P* < 0.04, Fig. 3D). No significant correlation was observed for other FCER1A SNPs in these populations analyzed (data not shown).

### Association of SNPs with sensitization to house dust mite

For comparaison, association analyses were also performed for allergic sensitization to house dust mites, as determined by positive specific IgE for dermatophygoides (Table5, Fig. 2C). Nine SNPs in or near eight genes showed significant association with allergic sensitization to house dust mite with *P* < 0.05. Interestingly, rs7851969 in *JAK2*, a top associated SNP with allergic sensitization, also showed the strongest association with the sensitization to house dust mites [OR (95% CI), 2.87 (1.56–5.26), *P* *=* 0.001]. In addition, three SNPs in 2 genes showed *P* < 0.01, including SNP rs6627 in *MAML1* [OR (95% CI), 0.57 (0.39–0.83), *P* *=* 0.004] and two SNPs in *NOD1* [rs5743334, OR (95% CI), 0.37 (0.18–0.75), *P* *=* 0.006; rs5743356, 0.36 (0.17–0.77), *P* *=* 0.008, respectively]. When the analysis was performed for black population; the associations with SNPs rs7851969 in gene *JAK2* [OR (95% CI), 3.08 (1.55–6.14), *P* *=* 0.001] and both SNPs in *NOD1* [rs5743334, OR (95% CI), 0.42 (0.19–0.90), *P* *=* 0.026; rs5743356, OR (95% CI), 0.33 (0.15–0.77), *P* *=* 0.010] were further confirmed.

**Table 5:** Association between genotyped single gene polymorphisms and house dust mite sensitization

rs ID	Gene	Chromosome	SNP	Minor allele frequency total		OR (95% CI)	*P*-value	Black non-sensitized (259) vs. sensitized (59)	
				Non-sensitized (N *=* 424)	Sensitized (N *=* 99)			OR (95% CI)	*P*-value
rs7851969	JAK2	9	C/G	0.05	0.11	2.87 (1.56–5.26)	0.001	3.08 (1.55–6.14)	0.001
rs6627	MAML1	5	G/A	0.38	0.27	0.57 (0.39–0.83)	0.004	0.74 (0.48–1.14)	0.741
rs5743334	NOD1	7	G/C	0.11	0.05	0.37 (0.18–0.75)	0.006	0.42 (0.19–0.90)	0.026
rs5743356	NOD1	7	T/G	0.09	0.05	0.36 (0.17–0.77)	0.008	0.33 (0.15–0.77)	0.010
rs12982518	FCER2	19	C/G	0.48	0.39	0.66 (0.48–0.93)	0.015	0.71 (0.47–1.07)	0.104
rs1058240	GATA3	10	A/G	0.22	0.13	0.58 (0.37–0.93)	0.025	0.58 (0.33–1.01)	0.053
rs6131034	CD40	20	C/G	0.05	0.11	2.05 (1.09–3.85)	0.026	1.41 (0.56–3.56)	0.470
rs163550	IL5RA	3	G/C	0.16	0.24	1.56 (1.04–2.31)	0.032	1.28 (0.73–2.24)	0.384
rs3749985	HLA-DPB1	6	G/C	0.07	0.02	0.31 (0.11–0.91)	0.033	0.38 (0.11–1.31)	0.125

OR, odds ratio; CI, confidence interval; all *P* values were analyzed under additive model adjusted for age, gender and race.

## Discussion

Previous studies have demonstrated that sensitization to cockroach allergen is one of the strongest risk factors for asthma. Although it is widely recognized that there is a genetic basis for allergen-induced sensitization, few studies have been performed to identify gene factors specifically for cockroach sensitization [8,19,30,31]. In this study, we have examined the association between allergic sensitization and SNPs in well-recognized candidate genes in the pathogenesis of allergic diseases. Our study populations were drawn from a prospective birth cohort consisting of allergens sensitized and non-sensitized individuals to aeroallergens. Thought it was birth cohort study, however, all selected individuals were greater than 5 months in age with average age (y) at 2.5 ± 2.07. Allergic sensitization was defined using a cut-point of 0.1 KU/L in the analyses, rather than a commonly used cutoff (0.35 KU/L), because we have previously shown no change for the direction and significance of the effects observed in analyses when either 0.1 or 0.35 KU/L was used as cutoffs [25]. Furthermore, genotyping data of a total of 895 SNPs in 179 genes are also available for those selected individuals. Those selected SNPs were either tagging SNPs or SNPs within the functional regions of gene or SNPs that were significantly associated with allergic diseases like asthma.

For this study, we selected *German cockroach, Dermatophagoides Pteronyssinus, Dermatophagoides Farinae, Alternaria Alternata, Cat Dander*, and *Dog Dander*, these most common indoor allergens for the tests for allergic sensitization. Our initial analysis was performed for allergic sensitization, which has an estimated heritability of 0.40–0.85 [32,33]. While many candidate genes have been previously reported for allergic sensitization, few have been firmly established. In our analysis, SNPs in seven selected candidate genes were associated with allergic sensitization, including several top ranking genes *JAK2, CNOT6*, and *MAML1*. Among these, SNP rs7851969 in *JAK2* showed consistent association with allergic sensitization, or specific sensitization to house dust mite or cockroach allergens. While these genes are functionally important in diverse diseases, none of the studied SNPs in these genes have been previously shown to be associated with allergic sensitization or other allergic diseases. For instance, *JAK2* are a family of tyrosine kinases that associated with cytokine receptors (e.g., *IL-3, IFN-gamma*). Upon receptor activation *JAKs* phosphorylate the transcription factors and initiate the *JAK-STAT* signaling pathway that is critical in the pathogenesis of allergic diseases like asthma [34]. *CNOT6* (*CCR4-NOT* transcription complex) belongs to a family of chemoattractant molecules involved in the directed migration of immune cells. *MAML1* (mastermind-like 1 (Drosophila)), as a protein-coding gene, is a novel modulator for *NF-κB* signaling and regulates cellular survival [35]. In addition, SNPs in or near *IL-4R, JAK1*, and *IL12B* showed modest association with allergic sensitization. These genes are well-known to be critical in mediating Th1/Th2 mediated immune responses, and SNPs in these genes have been associated with allergic diseases like asthma [36–39]. More importantly, very recent meta-analysis of genome wide association studies have identified ten loci that are associated with allergic sensitization [40], including SNPs in or near *TLR6, C11orf30, STAT6, SLC25A46, HLA-DGB1, IL1RL1, LPP, MYC, IL2* and *HLA-B*. While SNPs in *TLR6*, *C11orf30, STAT6, IL1RL1*, and *IL2* were also included in our dataset for analysis, none of SNPs in these genes showed significant association with allergic sensitization. In contrast, our study suggests several novel genes for allergic sensitization, such as *JAK2* and *CNOT6*.

We next performed an analysis on sub-groups, specifically those who are sensitized to cockroach allergen, one of our major focuses. It is well-known that sensitization to cockroach allergens is one of the strongest identified risks for greater asthma morbidity in low-income urban population [41,42]. Several genes (e.g., *HLA-DR, TSLP, MBL2*, and *IL-12A*) have been reported to be associated with different cockroach sensitization-related phenotypes [19,43,44]. In this analysis, 16 SNPs showed association with cockroach allergen sensitization, including top-ranking SNPs in several genes (*JAK1, JAK3, IL5RA, FCER1A*, and *ADAM33*) and genes with at least two associated SNPs (*JAK1, IL5RA, FCERA1, IL13*, and *CD86*). Of these, associations with SNPs in or near *JAK1, JAK3, FCER1A, ADAM33, IL13*, and *CD86* were further validated when analysis was limited to black population. When haplotype analysis was performed for several genes with multiple-associated SNPs, multiple haplotypes in FCER1A centered on SNP rs6665683 showed significant association with cockroach sensitization in total and black population only. It is known that FCER1A plays an important role in the development of allergy diseases [45]. Previous studies have suggested the linkage of two FCER1A functional polymorphisms −66T>C (rs2251746) and −315C>T (rs2427827) to allergic disorder [46], and total serum IgE levels [47,48]. SNP rs2427827, next to the promoter SNP rs2251746 in *FCER1A*, affects *FCER1A* transcriptional activity/FceRIa expression in mast cells and/or basophils [48,49]. In contrast, few haplotypes showed association with all allergen sensitization or particularly with sensitization to house dust mites features (data not shown), suggesting that cockroach sensitization may have unique features.

Interestingly, we found a correlation between the associated SNP rs6665683 and the levels of cockroach allergen specific IgE, and the effect on sIgE levels is allele dose dependent among all sensitized subjects with TT genotype having higher sIgE levels. The extremely higher levels of sIgE were also observed in the TT genotype of the non-black subpopulation, but not in the black population. The difference in two sub-populations may be due to (1) the source of SIgE and (2) population stratification. For the source of sIgE, it is suggested that some of those may be from maternofetal transfusion of IgE for these younger infants. However, maternal IgE does not cross the placenta [50] and the fetus is capable of producing IgE during the 11th week of gestation [51], most IgE detected in cord blood is likely to be produced by the fetus itself. Although a small amount of maternal blood might enter the fetal circulation during pregnancy and delivery [52], these will be completely replaced by the blood and allergen specific IgE produced by infants themselves. Further evidence by Kamemura et al. [53] demonstrated that IgE antibodies in CB are of fetal origin by using a novel allergen microarray of diamond-like-carbon-coated chip. Furthermore, in an *ex vivo* placental perfusion model, transfer of IgE-allergen complexes has been described for food allergen, but inhalant allergens were not transferred [54]. To control for potential confounding caused by population stratification, we genotyped ancestry informative markers for the estimation of ancestral proportion, and then used the estimated ancestral proportion as a covariate for the race variable in regression analysis.

For comparison, we also investigated the association of SNPs with allergic sensitization to house dust mites, one of the most common allergens. Among all those associated SNPs with house dust mites, several of them showed association with allergic sensitization, one SNP rs7851969 in *JAK2* showed association with both house dust mite and cockroach sensitization, suggesting that sensitization to house dust mite and cockroach allergens may share some of genetic features, which may be due to that (1) some of study subjects who are allergic multiple allergens, and (2) cross reactivity between allergens. Indeed, as shown in Table S3, among all these sensitized subjects, 48% of them are allergic to one allergen, 22.7% allergic to two allergens, 12.1% are allergic to three allergens, and <10% of subjects are allergic to more than 4, or 5 or 6 allergens.

In this study, there was a significant difference for the age between sensitized and non-sensitized subjects. Age and environmental factors may affect the allergen sensitization. To control for the possible confounding by age, our analysis was performed by using age as a covariate in logistic regression analysis. In the future, a longitudinal study may be essential to confirm whether the associations hold true over long periods of time in an age-matched population.

We recognize that, in this study, none of SNPs reached statistical significance after Bonferroni correction for multiple testing. It is widely recognized that the Bonferroni correction offers the most conservative approach to control for false positive and may conceal important functional variants. The overall modest *P* values seen in this study are more likely to be due to the modest sample size rather than a type-I error I (false positive). In addition, we have included multiple populations including Black/African, White, Hispanic, Cap Verdean, Asian/Pacific Islander, Caribbean, and unknown. Among these, the most common races are Black (62.1%), Hispanic (21.7%), and White (5.1%), and Cap Verdean (5.1%). Although it may be preferable to remove some races with too low-sample sizes from the analyses, we still included these in order to increase the total sample size to strengthen the power for data analysis.

In summary, we performed association analysis aiming at identifying genetic determinants for allergic sensitization, specifically cockroach sensitization. SNPs in or near *JAK2, CNOT6, MAML1, CD40, IL-4R*, and *IL-5RA* genes were associated with allergic sensitization and SNPs in or near *JAK1, JAK3*, *IL5RA, FCER1A*, and *ADAM33* genes were associated with cockroach sensitization. Specifically, SNP rs6665683 in *FCER1A* was associated with cockroach sensitization and levels of sIgE to cockroach allergen. Furthermore, SNP rs7851969 in *JAK2* showed consistent association with allergic sensitization, sensitization to HDM and cockroach allergen. Future studies will target on these genes to replicate the findings in larger samples of diverse ethnic populations, and subsequently investigate their biological relevance to disease.

## Supporting Information

**Table S1.** List of selected genes, SNPs, location and predicted function. **Table S2.** Clinical information. **Table S3.** Children with one or multiple (2–6) allergens.
