# Supplementary material for: Genetic determinants in the development of sensitization to environmental allergens in early childhood
Source: Immun Inflamm Dis. 2014 Nov 20;2(3):193–204. doi: 10.1002/iid3.38 (PMC4257764; doi:10.1002/iid3.38)
Supplement: Supplementary file 1 — Table S1. List of selected genes, SNPs, location and predicted function. Table S2. Clinical information. Table S3. Children with one or multiple (2–6) allergens. [file iid30002-0193-sd1.pdf]

Table S1. List of selected genes, SNPs, location and predicted function

SNP	SNP ID	Alleles	Gene	Chr	Position	Regulome DB Score	Feature
1	rs10047107	G/T	FCER1A	1	157527883	No Data	intron[NM_002001.2]
2	rs10052211	A/C	SPINK5	5	147434334	No Data	intron[NM_006846.2]
3	rs10062167	C/T	SPINK5	5	147489132	No Data	intron[NM_006846.2]
4	rs1007636	C/T	HLA-DMB	6	33012019	No Data	intron[NM_002118.3]
5	rs1008723	G/T	GSDML	17	35319793	6	intron[NM_018530.2]
6	rs10121491	C/T	JAK2	9	5036935	6	intron[NM_004972.2]
7	rs10169916	G/T	ALOX15	2	113320199	1f	Downstream
8	rs10192036	A/C	IL1RL1	2	102334643	5	missense[NM_016232.4]
9	rs10202402	C/G	DPP10	2	116317944	No Data	utr-3[NM_020868.2]
10	rs10205485	A/T	SDC1	2	20267275	5	missense[NM_002997.4];missense[NM_001006946.1];reference[NM_001006946.1]
11	rs10220622	C/T	CMA1	14	24046810	5	intron[NM_001836.2]
12	rs1024610	A/T	CCL2	17	29604344	4	
13	rs10267935	A/C	NPSR1	7	34852028	5	intron[NM_207172.1]
14	rs1026916	A/G	STAT3	17	37783361	No Data	intron[NM_213662.1]; intron[NM_139276.2]
15	rs10271556	C/T	NAMPT	7	105686110	6	intron[NM_005746.2]
16	rs1036199	A/C	HAVCR2	5	156464314	No Data	reference[NM_032782.3]
17	rs10399805	A/G	CHI3L1	1	201422621	1f	near-gene-5[NM_001276.2]
18	rs10399931	C/T	CHI3L1	1	201422703	1f	near-gene-5[NM_001276.2]
19	rs1041973	A/C	IL1RL1	2	102321900	5	missense[NM_003856.2];missense[NM_016232.4];reference[NM_016232.4]
20	rs1042337	C/T	HLA-DMB	6	33012959	No Data	reference[NM_002118.3]
21	rs1042720	A/G/T	ADRB2	5	148187826	4	coding-synonymous[NM_000024.4]
22	rs1043261	C/T	IL17RB	3	53874316	6	reference[NM_018725.3];near-gene-3[NM_022899.3]
23	rs10488854	A/G	GC	4	72842999	6	intron[NM_000583.2]
24	rs10491652	A/G	JAK2	9	5099531	5	intron[NM_004972.2]
25	rs10507391	A/T	ALOX5AP	13	30210096	5	intron[NM_001629.2]
26	rs1050900	A/T	HNMT	2	138488388	No Data	utr-3[NM_006895.2]
27	rs10515746	A/C	HAVCR2	5	156469146	5	near-gene-5[NM_032782.3]
28	rs1051594	C/T	MS4A1	11	59994630	6	utr-3[NM_152866.2]
29	rs10515944	A/C	CD28	2	204295514	4	intron[NM_006139.1]
30	rs1051740	C/T	EPHX1	1	224086256	5	missense[NM_000120.2]
31	rs1051741	C/T	EPHX1	1	224098852	4	reference[NM_000120.2]; near-gene-3[NM_014698.1]
32	rs1057141	A/G	TAP1	6	32926752	6	reference[NM_000593.5]
33	rs1058240	A/G	GATA3	10	8156604	5	utr-3[NM_002051.2]
34	rs1061622	G/T	TNFRSF1B	1	12175542	1f	missense[NM_001066.2]
35	rs1061624	A/G	TNFRSF1B	1	12189852	5	utr-3[NM_001066.2]
36	rs1063478	C/T	HLA-DMA	6	33025522	No Data	missense[NM_006120.2]
37	rs2427837	A/G	FCER1A	1	157525169	No Data	near-gene-5[NM_002001.2]
38	rs10751	C/T	HLA-DMB	6	33010561	No Data	utr-3[NM_002118.3]

39	rs10752126	C/G	GATA3	10	8146753	4	intron[NM_002051.2]
40	rs1075623	C/T	IL4R	16	27238438	No Data	intron[NM_001008699.1]
41	rs1077861	A/T	NOD2	16	49317048	6	intron[NM_022162.1]
42	rs10781522	A/G	TRAF2	9	138934874	5	intron[NM_021138.3]
43	rs10783218	C/T	VDR	12	46559010	No Data	intron[NM_000376.2]
44	rs10789166	A/G	JAK1	1	65085401	5	intron[NM_002227.2]
45	rs10815148	A/T	JAK2	9	5047284	5	intron[NM_004972.2]
46	rs10815160	G/T	JAK2	9	5106616	6	intron[NM_004972.2]
47	rs10817704	A/G	TNC	9	116847901	No Data	intron[NM_002160.2]
48	rs10856838	A/T	TLR10	4	38453568	1f	coding-synonymous[NM_030956.2]
49	rs10870140	C/T	TRAF2	9	138916240	2b	intron[NM_021138.3]
50	rs10889502	C/G	JAK1	1	65152570	5	intron[NM_002227.2]
51	rs10905277	A/G	GATA3	10	8137374	4	utr-5[NM_002051.2]
52	rs10932017	C/T	CD28	2	204286150	No Data	intron[NM_006139.1]
53	rs10940495	A/G	IL6ST	5	55298417	No Data	intron[NM_002184.2]
54	rs10974939	G/T	JAK2	9	5046482	6	intron[NM_004972.2]
55	rs10983756	C/T	TLR4	9	119513001	No Data	intron[NM_138554.2]
56	rs11079339	A/G	EPX	17	53625441	1f	reference[NM_000502.2]
57	rs11079786	A/G	LOC678655	17	43160915	5	-
58	rs11168293	G/T	VDR	12	46579983	6	intron[NM_000376.2]
59	rs11172106	C/G	GPR108	12	55799142	3a	-
60	rs11208534	A/G	JAK1	1	65115654	5	intron[NM_002227.2]
61	rs11208537	A/T	JAK1	1	65156220	5	intron[NM_002227.2]
62	rs11208545	G/T	JAK1	1	65171469	5	intron[NM_002227.2]
63	rs11208549	A/G	JAK1	1	65175697	No Data	intron[NM_002227.2]
64	rs11241090	A/G	TSLP	5	110437565	5	intron[NM_138551.2]
65	rs11260014	C/G	FCER2	19	7665715	4	intron[NM_002002.3]
66	rs1126579	C/T	IL8RB	2	218708979	6	utr-3[NM_001557.2]
67	rs1129055	A/G	CD86	3	123321009	No Data	missense[NM_006889.3];missense[NM_175862.3];reference[NM_175862.3]
68	rs1131535	A/G	TNFSF10	3	173706769	5	utr-3[NM_003810.2]
69	rs1131882	A/G	TBXA2R	19	3546923	5	coding-synonymous[NM_001060.4]
70	rs1134597	A/G	NLRC3	16	3529111	6	utr-3[NM_178844.2]
71	rs1135216	A/G	TAP1	6	32922953	4	reference[NM_000593.5]
72	rs11421	C/T	FCER1G	1	159455560	1f	utr-3[NM_004106.1]
73	rs1143627	C/T	IL1B	2	113310858	1b	near-gene-5[NM_000576.2]
74	rs1143633	A/G	IL1B	2	113306938	No Data	intron[NM_000576.2]
75	rs1143634	C/T	IL1B	2	113306861	5	reference[NM_000576.2]
76	rs1143643	A/G	IL1B	2	113304773	6	intron[NM_000576.2]
77	rs11465350	C/T	CCL26	7	75236704	6	near-gene-3[NM_006072.4]
78	rs11465353	G/T	CCL26	7	75236585	No Data	near-gene-3[NM_006072.4]
79	rs11465527	C/T	GPR108	6	52219379	6	-
80	rs11465545	C/G	IL17F	6	52215864	No Data	intron[NM_052872.3]

81	rs11466311	C/T	B9D2	19	46553588	5	intron[NM_030578.2]
82	rs11466741	C/T	TSLP	5	110436604	5	near-gene-5[NM_138551.2]
83	rs11466744	G/T	TSLP	5	110437865	5	intron[NM_033035.3]
84	rs11466749	A/G	TSLP	5	110440484	No Data	utr-3[NM_138551.2]
85	rs11466750	A/G	TSLP	5	110440793	6	utr-3[NM_033035.3]
86	rs11536887	A/G	TLR4	9	119517495	6	utr-3[NM_138554.2]
87	rs11553746	C/T	ACP1	2	262203	5	missense[NM_001040649.1]
88	rs11557467	G/T	ZPBP2	17	35282160	5	missense[NM_198844.2]
89	rs11567933	A/T	GATA3	10	8153086	5	intron[NM_002051.2]
90	rs11569304	A/C	CD40	20	44180717	4	intron[NM_152854.2]
91	rs11569323	C/T	CD40	20	44185711	No Data	intron[NM_001250.4]
92	rs11569562	C/T	C3	19	6629753	4	intron[NM_000064.2]
93	rs11574141	C/G	VDR	12	46521297	5	near-gene-3[NM_001017535.1]
94	rs11574845	G/T	NFKB2	10	104146373	4	intron[NM_002502.3];intron[NM_001077494.1]
95	rs11574849	A/G	NFKB2	10	104149686	2b	intron[NM_002502.3];intron[NM_001077494.1]
96	rs11575812	C/T	IL2	4	123590499	6	near-gene-3[NM_000586.3]
97	rs11587213	A/G	FCER1G	1	159451499	No Data	near-gene-5[NM_004106.1]
98	rs11650354	C/T	TBX21	17	43177091	1f	intron[NM_013351.1]
99	rs11652709	C/G	EPX	17	53626093	5	missense[NM_000502.2]
100	rs11657479	C/T	TBX21	17	43177900	5	utr-3[NM_013351.1]
101	rs11681540	A/G	DPP10	2	116132254	6	intron[NM_001004360.2]
102	rs11713419	A/G	IL5RA	3	3125267	6	utr-5[NM_175728.1]
103	rs11717893	C/T	CD86	3	123289515	No Data	intron[NM_006889.3]
104	rs11739089	C/T	CNOT6	5	179938020	6	near-gene-3[NM_015455.3]
105	rs11788963	A/C	JAK2	9	5110157	6	intron[NM_004972.2]
106	rs1181388	C/T	CD28	2	204284196	5	intron[NM_006139.1]
107	rs1181390	A/C	CD28	2	204280922	No Data	intron[NM_006139.1]
108	rs11864220	A/G	IL4R	16	27253240	No Data	intron[NM_001008699.1]
109	rs11883722	A/G	ICOS	2	204509090	No Data	near-gene-5[NM_012092.2]
110	rs11889031	C/T	DEFB1	2	204507639	6	-
111	rs11916344	C/T	C3orf1	3	120725842	No Data	near-gene-3[NM_016589.3]
112	rs11967235	C/T	HLA-DOB	6	32889411	5	intron[NM_002120.3]
113	rs12005893	C/G	JAK2	9	5068046	No Data	intron[NM_004972.2]
114	rs12005968	G/T	JAK2	9	5116542	No Data	intron[NM_004972.2]
115	rs12031995	C/T	JAK1	1	65165333	6	intron[NM_002227.2]
116	rs12063205	A/G	JAK1	1	65144640	No Data	intron[NM_002227.2]
117	rs12066872	C/T	CHRM3	1	237955156	6	intron[NM_000740.2]
118	rs1208	A/G	NAT2	8	18302596	6	missense[NM_000015.2]
119	rs12083537	A/G	IL6R	1	152647727	2a	intron[NM_181359.1]
120	rs12094497	A/G	FCER1G	1	159452927	5	intron[NM_004106.1]
121	rs12100565	A/T	CMA1	14	24044760	5	intron[NM_001836.2]
122	rs12135788	G/T	FCER1A	1	157531167	6	intron[NM_002001.2]

123	rs12201582	A/C	IL17F	6	52212648	No Data	intron[NM_052872.3]
124	rs12339666	G/T	JAK2	9	5053296	No Data	intron[NM_004972.2]
125	rs12368672	C/G	IL12RB1	12	55798737	6	-
126	rs12401767	A/G	IL6	1	111631320	4	-
127	rs12409333	A/G	JAK1	1	65190427	3a	intron[NM_002227.2]
128	rs1243963	C/T	GATA3	10	8152248	5	intron[NM_002051.2]
129	rs12448257	A/G	NLRC3	16	3539656	1f	intron[NM_178844.2]
130	rs12469506	C/T	IL1RL1	2	102332303	6	intron[NM_016232.4]
131	rs1254598	A/G	PTGER2	14	51850945	4	utr-5[NM_000956.2]
132	rs12563017	C/T	JAK1	1	65154449	4	intron[NM_002227.2]
133	rs12602891	C/T	EPX	17	53625120	4	utr-5[NM_000502.2]
134	rs12603332	C/T	ORMDL3	17	35336333	1f	intron[NM_139280.1]
135	rs12619285	A/G	CMA1	2	213532290	4	-
136	rs12652757	A/C	CMA1	5	148194422	No Data	-
137	rs12693993	A/G	CD28	2	204303842	No Data	intron[NM_006139.1]
138	rs1269486	A/G	GATA3	10	8136205	4	near-gene-5[NM_002051.2]
139	rs12698509	C/T	VKORC1L1	7	65056311	6	intron[NM_173517.3]
140	rs12709426	A/G	ACE	17	58915488	5	near-gene-5[NM_152830.1];missense[NM_000789.2]
141	rs12721583	A/T	STAT3	17	37743284	2a	intron[NM_213662.1]; intron[NM_139276.2]
142	rs12730021	A/G	JAK1	1	65189597	4	intron[NM_002227.2]
143	rs12743599	C/T	JAK1	1	65140630	No Data	intron[NM_002227.2]
144	rs12936231	C/G	ZPBP2	17	35282646	1f	intron[NM_199321.2]
145	rs12948058	A/G	CCL11	17	29634980	6	near-gene-5[NM_002986.2]
146	rs1295687	C/G	IL13	5	132022361	5	intron[NM_002188.2]
147	rs12980031	G/T	FCER2	19	7670436	2b	intron[NM_002002.3]
148	rs12982518	C/G	FCER2	19	7664316	5	intron[NM_002002.3]
149	rs12984870	A/G	FCER2	19	7670157	4	intron[NM_002002.3]
150	rs13013349	C/T	CMA1	2	113316797	6	-
151	rs13032029	C/T	CMA1	2	113316886	6	-
152	rs1304037	A/G	IL1A	2	113248707	5	utr-3[NM_000575.3]
153	rs13071247	A/C	CD80	3	120749483	6	intron[NM_005191.3]
154	rs13117745	C/T	NFKB1	4	103697738	No Data	intron[NM_003998.2]
155	rs1319313	A/G	NAMPT	7	105709759	5	intron[NM_005746.2]
156	rs1319501	C/T	NAMPT	7	105712989	2c	near-gene-5[NM_005746.2]
157	rs1321694	A/T	CLCA1	1	86720563	1f	reference[NM_001285.3]
158	rs13238709	C/T	IL4R	7	100570456	4	-
159	rs1327493	C/G	JAK2	9	4983082	5	intron[NM_004972.2]
160	rs1330363	A/G	TNC	9	116853811	No Data	intron[NM_002160.2]
161	rs13340504	C/T	CCL24	7	75277632	No Data	near-gene-3[NM_002991.2]
162	rs13380741	A/G	NOD2	16	49300439	5	intron[NM_022162.1]
163	rs13421193	A/G	DPP10	2	116265145	No Data	missense[NM_001004360.2];missense[NM_020868.2];reference[NM_020868.2]
164	rs13431828	C/T	IL1RL1	2	102321085	3a	utr-5[NM_003856.2]

165	rs1352844	C/T	GC	4	72866613	6	intron[NM_000583.2]
166	rs1353939	A/G	NOS1	12	116159736	5	intron[NM_000620.1]
167	rs1370128	C/T	IRAK3	12	64904905	No Data	intron[NM_007199.1]
168	rs1383258	A/G	HLA-DOB	6	32891383	5	intron[NM_002120.3]
169	rs1388604	A/T	NOD1	3	46251882	5	-
170	rs13900	C/T	CCL2	17	29608024	6	utr-3[NM_002982.3]
171	rs1399180	C/T	GATA3	10	8138725	5	intron[NM_002051.2]
172	rs1417938	A/T	CRP	1	157950810	6	intron[NM_000567.2]
173	rs1420101	A/G	IL1RL1	2	102324148	5	intron[NM_003856.2]
174	rs1422993	G/T	SPINK5	5	147484013	6	intron[NM_006846.2]
175	rs1431403	C/T	HLA-DPB1	6	33155009	1f	intron[NM_002121.4]
176	rs1446495	C/T	DPP10	2	116242430	No Data	missense[NM_001004360.2];missense[NM_020868.2];reference[NM_020868.2]
177	rs1485332	C/G	CD80	3	120757611	5	intron[NM_005191.3]
178	rs1491709	C/T	GC	4	72832430	No Data	intron[NM_000583.2]
179	rs1497056	C/T	JAK1	1	65202439	5	intron[NM_002227.2]
180	rs1497057	C/G	JAK1	1	65202562	5	intron[NM_002227.2]
181	rs151719	A/G	HLA-DMB	6	33011878	1f	intron[NM_002118.3]
182	rs1536798	A/C	JAK2	9	5046931	5	intron[NM_004972.2]
183	rs1538584	A/G	TJP2	9	71011963	No Data	intron[NM_201629.1]
184	rs1544325	A/G	COMT	22	18311668	4	intron[NM_000754.2]
185	rs1544410	A/G	VDR	12	46526102	5	intron[NM_000376.2]
186	rs1553316	C/T	HAVCR1	5	156412087	4	missense[NM_012206.2]
187	rs1556560	A/G	JAK1	1	65146174	6	intron[NM_002227.2]
188	rs1558068	A/T	NOD1	7	30469888	4	intron[NM_006092.1]
189	rs1609851	C/G	SPINK5	5	147425216	6	intron[NM_006846.2]
190	rs1624395	A/G	IRAK3	12	64904483	6	intron[NM_007199.1]
191	rs1635278	A/G	CCL16	17	31326324	No Data	near-gene-3[NM_004590.2]
192	rs163546	C/T	IL5RA	3	3098326	6	intron[NM_175726.1]
193	rs163549	A/C	IL5RA	3	3102730	4	intron[NM_175726.1]
194	rs163550	C/G	IL5RA	3	3101727	4	intron[NM_175726.1]
195	rs163913	C/T	HLA-DRB1	19	6673635	1f	-
196	rs16840252	C/T	CTLA4	2	204439764	No Data	near-gene-5[NM_005214.3]
197	rs16845759	G/T	TNFSF10	3	173715474	6	missense[NM_003810.2]
198	rs16847019	A/G	GC	4	72857866	5	intron[NM_000583.2]
199	rs16851435	G/T	ZBTB38	3	142644875	6	missense[NM_001080412.2]
200	rs16858811	G/T	IL8RA	2	218738088	No Data	missense[NM_000634.2]
201	rs16871026	A/G	TAP1	6	32923466	No Data	intron[NM_000593.5]
202	rs16896163	C/T	PTCRA	6	42994373	4	intron[NM_138296.2]
203	rs16947078	A/G	CCL2	17	43180499	6	-
204	rs1695	A/G	GSTP1	11	67109265	4	reference[NM_000852.2]
205	rs16965388	A/G	GSDML	17	35315665	6	reference[NM_018530.2];reference[NM_001042471.1];missense[NM_001042471.1]
206	rs16971620	A/C	CCL5	17	31226781	2b	intron[NM_002985.2]

207	rs17026703	A/T	IL5RA	3	3115785	No Data	intron[NM_175726.1]
208	rs17030	A/G	C3	19	6628989	4	reference[NM_000064.2]
209	rs17032705	A/G	NFKB1	4	103652004	6	intron[NM_003998.2]
210	rs17032740	A/G	NFKB1	4	103663809	5	intron[NM_003998.2]
211	rs17032779	C/T	NFKB1	4	103685279	5	intron[NM_003998.2]
212	rs17042905	C/T	CCL2	2	113584427	6	-
213	rs17107730	C/G	SPINK5	5	147460001	No Data	intron[NM_006846.2]
214	rs17125377	A/T	CCL2	14	51872254	No Data	-
215	rs17127063	A/G	JAK1	1	65084930	4	reference[NM_002227.2]
216	rs17127114	A/G	JAK1	1	65108419	No Data	intron[NM_002227.2]
217	rs17127117	C/T	JAK1	1	65111363	No Data	intron[NM_002227.2]
218	rs17127175	C/T	JAK1	1	65187448	2b	intron[NM_002227.2]
219	rs17159836	C/T	FCER2	19	7663908	5	intron[NM_002002.3]
220	rs17159840	C/T	FCER2	19	7664194	1f	intron[NM_002002.3]
221	rs17191234	A/C	CCL5	6	32672659	6	-
222	rs17281995	C/G	CD86	3	123322331	No Data	utr-3[NM_006889.3]
223	rs17425819	C/T	JAK2	9	5104773	No Data	intron[NM_004972.2]
224	rs17533594	A/G	CD28	2	204289440	6	intron[NM_006139.1]
225	rs17561	G/T	IL1A	2	113253694	3a	reference[NM_000575.3]
226	rs17564816	A/G	TNFSF13B	13	107725504	No Data	intron[NM_006573.3]
227	rs17576	A/G	MMP9	20	44073632	5	reference[NM_004994.2]
228	rs17659401	A/G	IL5RA	3	3087865	No Data	intron[NM_175726.1]
229	rs17778257	A/T	ADRB2	5	148184770	6	near-gene-5[NM_000024.4]
230	rs17852635	A/G	IL12RB1	19	18047575	No Data	coding-synonymous[NM_005535.1]
231	rs17857295	C/G	VISA	20	3786441	No Data	reference[NM_020746.2]
232	rs17861084	G/T	CYP1A1	15	72799072	No Data	utr-3[NM_000499.2]
233	rs17884458	C/T	IL5RA	3	3122196	5	intron[NM_175728.1]
234	rs1799929	C/T	NAT2	8	18302274	No Data	reference[NM_000015.2]
235	rs1799964	C/T	LTA	6	31650287	4	near-gene-3[NM_000595.2]
236	rs1800454	A/G	TAP2	6	32908390	6	missense[NM_018833.2]; missense[NM_000544.3]; reference[NM_000544.3]
237	rs1800471	C/G	TGFB1	19	46550716	4	near-gene-3[NM_030578.2]; reference[NM_000660.3]
238	rs1800587	C/T	IL1A	2	113259431	5	utr-5[NM_000575.3]
239	rs1800621	A/G	TNFRSF1B	1	12191158	4	utr-3[NM_001066.2]
240	rs1800630	A/C	TNF	6	31650455	2b	near-gene-5[NM_000594.2]
241	rs1800779	A/G	NOS3	7	150320876	4	intron[NM_000603.3]
242	rs1800875	A/G	CCL3	14	24049178	6	-
243	rs1800896	A/G	DLGAP3	1	205013520	6	-
244	rs1800925	C/T	IL13	5	132020708	2b	near-gene-5[NM_002188.2]
245	rs1801275	A/G	IL4R	16	27281901	No Data	reference[NM_000418.2]
246	rs1801279	A/G	NAT2	8	18301984	5	missense[NM_000015.2]
247	rs1801280	C/T	NAT2	8	18302134	5	missense[NM_000015.2]

248	rs1805010	A/C/G/T	IL4R	16	27263704	5	reference[NM_001008699.1]
249	rs1805016	G/T	IL4R	16	27282428	4	missense[NM_000418.2]
250	rs1805017	A/G	PLA2G7	6	46792181	No Data	missense[NM_005084.2]
251	rs1821777	C/T	IRAK3	12	64892495	6	intron[NM_007199.1]
252	rs1823035	C/T	CYFIP2	5	156680247	6	missense[NM_014376.2]
253	rs1858480	A/G	LOC100128	1	150568359	5	intron[XM_001718271.1]
254	rs1860189	C/T	ZPBP2	17	29602471	5	-
255	rs1861494	C/T	IFNG	12	66837676	4	intron[NM_000619.2]
256	rs1861757	A/G	NOD2	16	49310316	2b	intron[NM_022162.1]
257	rs1861758	C/T	NOD2	16	49309288	1f	intron[NM_022162.1]
258	rs1862442	A/G	SPINK5	5	147469463	6	intron[NM_006846.2]
259	rs1878672	C/G	IL10	1	205010336	5	intron[NM_000572.2]
260	rs1879877	A/C	CD28	2	204278245	5	near-gene-5[NM_006139.1]
261	rs1881457	A/C	IL13	5	132020308	3a	near-gene-5[NM_002188.2]
262	rs1882200	C/T	IRAK3	12	64883968	4	intron[NM_007199.1]
263	rs1889570	A/G	ORMDL3	6	52218693	No Data	-
264	rs1894406	A/G	HLA-DOB	6	32895034	6	-
265	rs1894411	A/G	TAP2	6	32900951	6	intron[NM_018833.2]
266	rs1921622	A/G	IL1RL1	2	102332499	6	intron[NM_016232.4]
267	rs1927912	A/G	TLR4	9	119509247	4	intron[NM_138554.2]
268	rs1933064	A/G	LOC100128 468	1	150568200	5	intron[XM_001718271.1]
269	rs1940475	C/T	MMP8	11	102098458	No Data	reference[NM_002424.1]
270	rs1946518	G/T	ORMDL3	11	111540668	5	Promoter
271	rs194675	A/T	HLA-DMB	6	33013724	6	intron[NM_002118.3]
272	rs1956918	C/T	STAT3	14	24050151	6	Promoter
273	rs1956922	A/G	LOC100133	14	24048014	6	near-gene-3[XM_001716996.1]
274	rs1978331	C/T	LTA4H	12	94933332	No Data	intron[NM_000895.1]
275	rs1989969	A/C/T	VDR	12	46564277	No Data	intron[NM_001017535.1]
276	rs2001750	A/G	CD86	3	123309207	No Data	intron[NM_006889.3]
277	rs2008188	C/G	GUSB	7	65066448	No Data	intron[NM_000181.2]
278	rs2031904	C/T	JAK2	9	5077087	No Data	intron[NM_004972.2]
279	rs2037955	G/T	NOD1	7	30478474	5	intron[NM_006092.1]
280	rs2053724	C/G	DPP10	2	116227287	No Data	missense[NM_020868.2];missense[NM_001004360.2];reference[NM_001004360.2]
281	rs20541	C/T	IL13	5	132023863	No Data	reference[NM_002188.2]
282	rs20544	C/T	LOC100128	20	44078417	6	intron[XM_001722009.1]
283	rs2056576	C/T	MMP9	7	22727727	No Data	Promoter
284	rs2057768	A/G	CD40	16	27229596	5	Promoter
285	rs2063979	A/G	CCL16	17	31327679	6	utr-3[NM_004590.2]
286	rs2067085	C/G	NOD2	16	49291360	4	reference[NM_022162.1]
287	rs2069714	A/C	IFNG	12	66837311	No Data	intron[NM_000619.2]
288	rs2069718	C/T	IFNG	12	66836429	No Data	intron[NM_000619.2]

289	rs2069743	A/G	IL13	5	132021174	6	near-gene-5[NM_002188.2]
290	rs2069744	C/T	IL13	5	132022568	5	intron[NM_002188.2]
291	rs2069763	G/T	IL2	4	123596932	5	reference[NM_000586.3]
292	rs2069776	C/T	IL2	4	123591426	5	near-gene-3[NM_000586.3]
293	rs2069838	C/T	IL6	7	22735004	No Data	intron[NM_000600.2]
294	rs2069849	C/T	IL6	7	22737681	5	reference[NM_000600.2]
295	rs2070120	C/T	HLA-DOB	6	32888892	6	utr-3[NM_002120.3]
296	rs2070121	C/T	HLA-DOB	6	32889532	No Data	reference[NM_002120.3]
297	rs2070874	C/T	IL4	5	132037609	2b	utr-5[NM_172348.1]
298	rs2070901	G/T	FCER1G	1	159451682	4	near-gene-5[NM_004106.1]
299	rs2070902	C/T	FCER1G	1	159454289	6	intron[NM_004106.1]
300	rs2071351	A/G	HLA-DPB1	6	33151908	4	intron[NM_002121.4]
301	rs2071354	C/T	HLA-DPB1	6	33152366	5	intron[NM_002121.4]
302	rs2071473	A/G	HLA-DOB	6	32890583	6	intron[NM_002120.3]
303	rs2071475	C/T	HLA-DOB	6	32890365	1f	intron[NM_002120.3]
304	rs2071481	A/G	TAP1	6	32927843	5	intron[NM_000593.5]
305	rs2071484	A/G	ACP5	19	11549460	1f	utr-5[NM_001611.3]
306	rs2071541	C/T	PSMB8	6	32920836	4	near-gene-5[NM_004159.4]
307	rs2071543	A/C	PSMB8 ; TAP1	6	32919607	4	missense[NM_148919.3];intron[NM_004159.4];near-gene-3[NM_000593.5]
308	rs2071554	A/G	HLA-DOB	6	32892654	4	missense[NM_002120.3]
309	rs2071556	A/C	HLA-DMB	6	33012579	1f	intron[NM_002118.3]
310	rs2072496	A/G	JAK3	19	17807054	5	intron[NM_000215.2]
311	rs2072915	A/T	RXRB	6	33270060	5	utr-3[NM_021976.3]
312	rs2073342	C/G	RNASE3	14	20430056	6	reference[NM_002935.2]
313	rs2074570	A/G	IL4R	16	27282658	5	utr-3[NM_000418.2]
314	rs2075632	C/T	CLCA1	1	86727179	6	intron[NM_001285.3]
315	rs2075818	C/G	NOD1	7	30462907	No Data	reference[NM_006092.1]
316	rs2075820	A/G	NOD1	7	30458762	5	missense[NM_006092.1]
317	rs2075821	A/G	NOD1	7	30457836	5	coding-synonymous[NM_006092.1]
318	rs2076752	A/G	NOD2	16	49288597	5	utr-5[NM_022162.1]
319	rs2104772	A/T	TNC	9	116848606	5	reference[NM_002160.2]
320	rs2107301	C/T	VDR	12	46541837	5	intron[NM_000376.2]
321	rs2107538	C/T	CCL5	17	31231893	4	near-gene-5[NM_002985.2]
322	rs2110586	C/T	JAK3	19	17818309	5	intron[NM_000215.2]
323	rs2111235	C/T	NOD2	16	49291470	2a	intron[NM_022162.1]
324	rs2115819	C/T	ALOX5	10	45221095	No Data	intron[NM_000698.2]
325	rs2145412	C/T	CLCA1	1	86711718	No Data	reference[NM_001285.3]
326	rs2169650	C/T	TBX21	11	67120493	6	Promoter
327	rs2184658	C/G	HLX	1	219119080	4	near-gene-5[NM_021958.2]
328	rs222014	A/G	GC	4	72851795	No Data	intron[NM_000583.2]
329	rs222017	A/T	GC	4	72854376	6	intron[NM_000583.2]

330	rs222040	C/T	GC	4	72835796	No Data	intron[NM_000583.2]
331	rs2227631	A/G	SERPINE1	7	100556258	1b	near-gene-5[NM_000602.2]
332	rs2227684	A/G	SERPINE1	7	100563651	1f	intron[NM_000602.2]
333	rs2228043	C/G	IL6ST	5	55287688	No Data	utr-3[NM_175767.1]; missense[NM_002184.2]
334	rs2228046	C/T	IL6ST	5	55286484	6	missense[NM_002184.2]; utr-3[NM_175767.1]
335	rs2228138	C/T	FCER2	19	7661056	5	reference[NM_002002.3]
336	rs2228396	A/G	TAP2	6	32905787	No Data	missense[NM_018833.2]; missense[NM_000544.3]; reference[NM_000544.3]
337	rs2228397	G/T	TAP2	6	32908202	6	coding-synonymous[NM_000544.3]
338	rs2229094	C/T	LTA	6	31648535	4	missense[NM_000595.2]
339	rs12136904	C/T	FCER1A	1	157532516	6	intron[NM_002001.2]
340	rs2230587	C/T	JAK1	1	65083850	6	reference[NM_002227.2]
341	rs2234671	C/G	IL8RA	2	218737353	No Data	missense[NM_000634.2]
342	rs2235834	C/G	PTCRA	6	42999183	5	intron[NM_138296.2]
343	rs2239347	G/T	IL4R	16	27266522	No Data	intron[NM_001008699.1]
344	rs2239704	G/T	LTA	6	31648120	1f	utr-5[NM_000595.2]
345	rs2240478	C/T	CCL26	7	75239354	5	intron[NM_006072.4]
346	rs2243248	G/T	IL4	5	132036543	No Data	near-gene-5[NM_000589.2]
347	rs2243253	C/T	IL4	5	132038660	4	intron[NM_172348.1]
348	rs2243261	G/T	IL4	5	132040705	4	intron[NM_172348.1]
349	rs2243263	C/G	IL4	5	132041198	2b	intron[NM_172348.1]
350	rs2243274	A/G	IL4	5	132042731	6	intron[NM_172348.1]
351	rs2243279	A/G	IL4	5	132044126	5	intron[NM_172348.1]
352	rs2243283	C/G	IL4	5	132044492	5	intron[NM_172348.1]
353	rs2244719	C/T	CYP24A1	20	52216265	5	intron[NM_000782.3]
354	rs2247119	C/T	PHF11	13	48985143	6	intron[NM_001040443.1]
355	rs2250889	C/G	MMP9	20	44075813	4	missense[NM_004994.2];near-gene-3[XM_001722009.1]
356	rs2256023	C/T	NOD1	7	30481516	No Data	intron[NM_006092.1]
357	rs2270418	G/T	TNFSF10	3	173723693	4	intron[NM_003810.2]
358	rs2274276	C/G	PHF11	13	48993954	6	intron[NM_001040444.1]
359	rs2274471	C/T	JAK2	9	4975879	4	intron[NM_004972.2]
360	rs2251746	C/T	FCER1A	1	157538684	5	intron[NM_002001.2]
361	rs2275254	C/T	CHIA	1	111663497	5	missense[NM_201653.2];missense[NM_021797.2];reference[NM_021797.2]
362	rs2275806	A/G	GATA3	10	8135346	4	utr-5[NM_207423.1];near-gene-5[NM_001002295.1]
363	rs2280094	A/C	ADAM33	20	3601709	5	intron[NM_153202.1]
364	rs2280789	C/T	CCL5	17	31231116	1f	intron[NM_002985.2]
365	rs2282290	A/G	CHIA	1	111664974	5	near-gene-3[NM_201653.2]
366	rs2284358	A/G	NOD1	7	30455532	6	intron[NM_006092.1]
367	rs2287772	C/T	SPINK5	5	147424978	6	intron[NM_006846.2]
368	rs2289277	C/G	TSLP	5	110436966	2b	intron[NM_033035.3]
369	rs2289278	C/G	TSLP	5	110437047	4	utr-5[NM_138551.2]
370	rs2290608	A/G	IL5RA	3	3126759	4	utr-5[NM_175726.1]
371	rs2290610	A/G	IL5RA	3	3114957	No Data	missense[NM_000564.2];reference[NM_175724.1];missense[NM_175724.1]

372	rs2291299	A/G	C17orf66	17	31215519	No Data	intron[NM_152781.2]
373	rs2293044	G/T	NOS1	12	116142374	No Data	coding-synonymous[NM_000620.1]
374	rs2293054	A/G	NOS1	12	116186097	No Data	coding-synonymous[NM_000620.1]
375	rs2296241	A/G	CYP24A1	20	52219626	4	reference[NM_000782.3]
376	rs2297660	A/C	LRP8	1	53504903	1f	coding-synonymous[NM_017522.3]
377	rs2298903	A/T	SELP	1	167834140	No Data	intron[NM_003005.3]
378	rs2301149	C/G	KCNMB1	5	169738534	5	missense[NM_004137.2]; intron[NM_001034838.1]
379	rs2302004	C/T	CCL24	7	75280791	5	intron[NM_002991.2]
380	rs2302006	A/C	CCL24	7	75280666	5	reference[NM_002991.2]
381	rs2302009	G/T	CCL26	7	75236934	5	utr-3[NM_006072.4]
382	rs2303071	A/G	SPINK5	5	147468560	6	reference[NM_006846.2]
383	rs230495	A/G	NFKB1	4	103706336	3a	intron[NM_003998.2]
384	rs230504	C/T	NFKB1	4	103700596	1f	intron[NM_003998.2]
385	rs2309428	A/C	TJP2	9	71032843	5	missense[NM_201629.1];missense[NM_004817.2];reference[NM_004817.2]
386	rs231775	A/G	CTLA4	2	204440959	No Data	reference[NM_005214.3];reference[NM_001037631.1];missense[NM_001037631.1]
387	rs231777	C/T	CTLA4	2	204441833	No Data	intron[NM_005214.3]
388	rs231778	A/G	CTLA4	2	204442066	No Data	intron[NM_001037631.1]
389	rs231779	C/T	CTLA4	2	204442732	5	intron[NM_005214.3]
390	rs2332096	G/T	CD86	3	123303833	3a	intron[NM_006889.3]
391	rs2344690	A/G	KCNS3	2	17963640	2b	intron[NM_002252.3]
392	rs23544	A/G	HLA-DMB	6	33011615	5	intron[NM_002118.3]
393	rs2364572	A/G	CHI3L1	1	201413209	4	near-gene-3[NM_001276.2]
394	rs2395269	G/T	TAP1	6	32925752	5	intron[NM_000593.5]
395	rs241423	C/T	TAP1	6	32925988	5	intron[NM_000593.5]
396	rs241424	C/T	TAP2	6	32912912	1f	intron[NM_018833.2]
397	rs241426	A/T	TAP2	6	32912531	No Data	intron[NM_018833.2]
398	rs241428	A/C	TAP2	6	32912048	5	intron[NM_018833.2]
399	rs241429	C/T	TAP2	6	32911818	6	intron[NM_018833.2]
400	rs241436	C/T	TAP2	6	32905854	6	intron[NM_018833.2]
401	rs241439	A/C	TAP2	6	32905515	4	intron[NM_018833.2]
402	rs241448	C/T	TAP2	6	32904663	1f	intron[NM_018833.2]; reference[NM_000544.3]
403	rs241453	C/T	TAP2	6	32904204	6	intron[NM_018833.2]
404	rs2416257	A/G	WDR36	5	110463389	No Data	intron[NM_139281.2]
405	rs25680	A/G	CD27	12	6424889	4	missense[NM_001242.4]
406	rs2569190	A/G	CD14	5	139993100	1b	utr-5[NM_000591.2]
407	rs25881	C/T	CSF2	5	131439037	5	intron[NM_000758.2]
408	rs25882	C/T	CSF2	5	131439359	5	missense[NM_000758.2]
409	rs25884	A/G	CSF2	5	131440137	3a	near-gene-3[NM_000758.2]
410	rs260547	C/G	HLA-DMB	6	33015256	6	intron[NM_002118.3]
411	rs2621321	C/T	TAP2	6	32897458	6	near-gene-3[NM_018833.2]
412	rs2621332	C/T	HLA-DOB	6	32888295	No Data	near-gene-3[NM_002120.3]
413	rs2621366	G/T	HLA-DOB	6	32876958	6	-

414	rs2664593	C/G	ALOX15	17	4491881	5	near-gene-5[NM_001140.3]
415	rs2670289	A/C	CD80	3	120757276	5	intron[NM_005191.3]
416	rs2681417	A/G	CD86	3	123307887	No Data	missense[NM_006889.3];missense[NM_175862.3];reference[NM_175862.3]
417	rs2681420	A/G	CD86	3	123316747	6	intron[NM_006889.3]
418	rs2681426	C/T	CD86	3	123283920	6	intron[NM_006889.3]
419	rs2702945	A/G	DEFB1	8	6722097	No Data	intron[NM_005218.3]
420	rs2709799	C/G	NOD1	7	30481428	1f	intron[NM_006092.1]
421	rs2709800	G/T	NOD1	7	30479296	5	intron[NM_006092.1]
422	rs272008	A/G	DPP10	2	116318101	No Data	utr-3[NM_020868.2]
423	rs2734705	A/G	CLCA1	1	86724912	No Data	reference[NM_001285.3]
424	rs2736726	A/G	NOD1	7	30485555	4	-
425	rs2738047	A/G	DEFB1	8	6715708	No Data	missense[NM_005218.3]
426	rs2738755	C/T	HLX	1	219124269	5	reference[NM_021958.2]
427	rs2741136	A/G	CCR3	8	6724606	No Data	Promoter
428	rs2744537	G/T	RXRB	6	33270193	1f	utr-3[NM_021976.3]
429	rs2749935	A/T	IL17F	6	131931574	No Data	Promoter
430	rs2753356	G/T	CLCA1	1	86736663	5	intron[NM_001285.3]
431	rs2762934	A/G	CYP24A1	20	52204668	4	utr-3[NM_000782.3]
432	rs2770146	A/G	TLR4	9	119513159	No Data	intron[NM_138554.2]
433	rs2779248	C/T	NOS2A	17	23151959	4	near-gene-5[NM_000625.3]
434	rs2780890	A/G	JAK1	1	65074078	5	intron[NM_002227.2]
435	rs2780894	C/T	JAK1	1	65081964	No Data	intron[NM_002227.2]
436	rs2781659	A/G	IL17F	6	131933513	No Data	Promoter
437	rs2781665	A/T	MED23	6	131934940	5	near-gene-3[NM_015979.2]
438	rs2787094	C/G	ADAM33	20	3597161	5	utr-3[NM_153202.1]
439	rs2791494	A/G	CLCA1	1	86731761	5	missense[NM_001285.3]
440	rs28408445	C/G	SPINK5	5	147483711	6	reference[NM_006846.2]
441	rs2853563	A/G	VDR	12	46522005	6	utr-3[NM_000376.2]
442	rs2853564	C/T	VDR	12	46564754	5	intron[NM_001017535.1]
443	rs2853694	A/C	IL12B	5	158681666	No Data	intron[NM_002187.2]
444	rs2856827	A/T	HLA-DPB1	6	33157252	4	intron[NM_002121.4]
445	rs2856837	C/T	IL1A	2	113258396	4	intron[NM_000575.3]
446	rs2856993	C/G	TAP2	6	32899381	No Data	intron[NM_018833.2]
447	rs2856997	G/T	HLA-DOB	6	32889754	6	intron[NM_002120.3]
448	rs2857114	C/T	HLA-DOB	6	32887974	6	near-gene-3[NM_002120.3]
449	rs2857656	C/G	CCL2	17	29606120	4	near-gene-5[NM_002982.3]
450	rs2890618	C/T	JAK2	9	4981840	No Data	intron[NM_004972.2]
451	rs28914820	A/G	CCL5	17	31221048	No Data	near-gene-3[NM_002985.2]
452	rs2906766	C/T	NOD1	7	30466100	2b	utr-5[NM_006092.1]
453	rs2907748	C/T	NOD1	7	30439548	5	intron[NM_006092.1]
454	rs2907749	A/G	NOD1	7	30452266	2b	intron[NM_006092.1]
455	rs2970498	C/T	NOD1	7	30444581	No Data	intron[NM_006092.1]

456	rs2970500	C/G	NOD1	7	30445010	No Data	intron[NM_006092.1]
457	rs2970501	C/T	NOD1	7	30462518	No Data	intron[NM_006092.1]
458	rs2975634	A/G	NOD1	7	30458218	5	missense[NM_006092.1]
459	rs3020208	C/T	NOD1	7	30458667	5	reference[NM_006092.1]
460	rs3024498	A/G	IL10	1	205008152	4	utr-3[NM_000572.2]
461	rs3024530	A/G	IL4R	16	27258188	6	intron[NM_001008699.1]
462	rs3024535	C/T	IL4R	16	27259622	5	intron[NM_001008699.1]
463	rs3024543	A/G	IL4R	16	27260731	5	intron[NM_001008699.1]
464	rs3024544	C/T	IL4R	16	27260858	5	intron[NM_001008699.1]
465	rs3024547	C/T	IL4R	16	27261862	No Data	intron[NM_001008699.1]
466	rs3024548	C/G	IL4R	16	27262032	No Data	intron[NM_001008699.1]
467	rs3024560	G/T	IL4R	16	27264168	6	intron[NM_001008699.1]
468	rs3024563	C/T	IL4R	16	27264242	6	intron[NM_001008699.1]
469	rs3024576	A/G	IL4R	16	27265691	5	intron[NM_001008699.1]
470	rs3024582	G/T	IL4R	16	27266695	No Data	intron[NM_001008699.1]
471	rs3024585	A/G	IL4R	16	27267345	6	intron[NM_001008699.1]
472	rs3024592	A/T	IL4R	16	27268433	No Data	intron[NM_001008699.1]
473	rs3024604	C/T	IL4R	16	27270731	3a	intron[NM_001008699.1]
474	rs3024613	C/T	IL4R	16	27271754	No Data	intron[NM_001008699.1]
475	rs3024614	A/G	IL4R	16	27271846	6	intron[NM_001008699.1]
476	rs3024620	C/G	IL4R	16	27272419	No Data	intron[NM_001008699.1]
477	rs3024622	C/G	IL4R	16	27272954	3a	intron[NM_001008699.1]
478	rs3024630	A/G	IL4R	16	27273627	No Data	intron[NM_001008699.1]
479	rs3024633	A/G	IL4R	16	27274000	No Data	utr-3[NM_001008699.1]
480	rs3024635	C/T	IL4R	16	27274370	6	near-gene-3[NM_001008699.1]
481	rs3024647	A/G	IL4R	16	27275473	No Data	intron[NM_000418.2]
482	rs3024658	A/G	IL4R	16	27277955	No Data	intron[NM_000418.2]
483	rs3024668	A/G	IL4R	16	27279450	No Data	intron[NM_000418.2]
484	rs3024669	A/C	IL4R	16	27279817	No Data	intron[NM_000418.2]
485	rs3024670	G/T	IL4R	16	27279896	No Data	intron[NM_000418.2]
486	rs3024672	A/G	IL4R	16	27280632	No Data	intron[NM_000418.2]
487	rs3024675	A/C	IL4R	16	27280968	No Data	intron[NM_000418.2]
488	rs3024676	A/C	IL4R	16	27281059	No Data	intron[NM_000418.2]
489	rs3024679	C/T	IL4R	16	27282571	No Data	coding-synonymous[NM_000418.2]
490	rs3024955	C/G	STAT6	12	55785421	5	intron[NM_003153.3]
491	rs3024957	C/G	STAT6	12	55784302	5	intron[NM_003153.3]
492	rs3024971	A/C	STAT6	12	55779994	No Data	intron[NM_003153.3]
493	rs3024974	C/T	STAT6	12	55779012	2b	intron[NM_003153.3]
494	rs3087243	A/G	CTLA4	2	204447164	3a	near-gene-3[NM_005214.3]
495	rs3087271	A/C	IL17F	2	113611671	6	Promoter
496	rs3091332	A/T	CCL2	17	29604933	No Data	near-gene-5[NM_002982.3]
497	rs3092932	C/T	CD40LG	X	135565013	No Data	intron[NM_000074.2]

498	rs3100701	C/T	HNMT	2	138446819	No Data	intron[NM_001024075.1]
499	rs310199	C/T	JAK1	1	65122710	3a	intron[NM_002227.2]
500	rs310207	A/G	JAK1	1	65119629	4	intron[NM_002227.2]
501	rs310227	A/G	JAK1	1	65095730	No Data	intron[NM_002227.2]
502	rs310235	A/G	JAK1	1	65107911	No Data	intron[NM_002227.2]
503	rs310236	C/G	JAK1	1	65106445	No Data	intron[NM_002227.2]
504	rs310241	C/T	JAK1	1	65075926	5	intron[NM_002227.2]
505	rs310244	C/T	JAK1	1	65078465	5	intron[NM_002227.2]
506	rs310247	C/T	JAK1	1	65079997	5	intron[NM_002227.2]
507	rs3116486	C/T	CD28	2	204293747	No Data	intron[NM_006139.1]
508	rs3116494	A/G	CD28	2	204300266	6	intron[NM_006139.1]
509	rs3132291	C/T	RXRA	9	136472739	5	near-gene-3[NM_002957.3]
510	rs3134996	A/T	HLA-DQB1	6	32744844	6	-
511	rs3135021	A/G	HLA-DPB1	6	33153536	6	intron[NM_002121.4]
512	rs3135499	A/C	NOD2	16	49323628	6	utr-3[NM_022162.1]
513	rs3136551	A/G	CD27	12	6424121	3a	near-gene-5[NM_001242.4]
514	rs3138060	C/G	IL8RA	2	218739745	5	intron[NM_000634.2]
515	rs3138118	C/T	CCL20	2	228390846	1b	near-gene-3[NM_004591.1]
516	rs315951	C/G	IL1RN	2	113607057	5	utr-3[NM_173843.1];utr-3[NM_173841.1];utr-3[NM_173842.1]
517	rs315952	C/T	IL1RN	2	113606775	No Data	coding-synonymous[NM_173843.1];coding-
518	rs3181100	C/G	CD28	2	204280251	No Data	intron[NM_006139.1]
519	rs3181113	G/T	CD28	2	204310155	6	utr-3[NM_006139.1]
520	rs3212227	A/C	IL12B	5	158675528	No Data	utr-3[NM_002187.2]
521	rs3212701	A/G	JAK3	19	17818024	5	intron[NM_000215.2]
522	rs3212711	C/T	JAK3	19	17816021	5	intron[NM_000215.2]
523	rs3212714	C/T	JAK3	19	17815947	5	intron[NM_000215.2]
524	rs3212723	A/C	JAK3	19	17815215	5	missense[NM_000215.2]
525	rs3212750	C/T	JAK3	19	17810204	5	intron[NM_000215.2]
526	rs3212752	A/G	JAK3	19	17809732	1f	intron[NM_000215.2]
527	rs3212756	A/C	JAK3	19	17808847	5	intron[NM_000215.2]
528	rs3212760	C/T	JAK3	19	17808546	5	intron[NM_000215.2]
529	rs3212799	A/G	JAK3	19	17798516	5	utr-3[NM_000215.2]
530	rs3213091	A/G	IL12B	5	158683818	5	intron[NM_002187.2]
531	rs324010	C/T	NAB2	12	55768519	1f	near-gene-5[NM_005967.3]
532	rs324013	C/T	STAT6	12	55796928	6	-
533	rs324015	A/G	STAT6	12	55776367	1f	utr-3[NM_003153.3]
534	rs324019	C/T	NAB2	12	55772914	1f	intron[NM_005967.3]
535	rs324957	A/G	NPSR1	7	34767897	3a	intron[NM_207173.1]
536	rs324981	A/T	NPSR1	7	34784638	6	reference[NM_207173.1];reference[NM_207172.1];missense[NM_207172.1]
537	rs334782	A/G	IL5RA	3	3123458	6	intron[NM_175727.1]
538	rs334807	G/T	IL5RA	3	3104349	5	intron[NM_175726.1]
539	rs335826	C/G	IL5RA	3	3118888	3a	intron[NM_175726.1]

540	rs339392	G/T	C3	19	6673022	1f	-
541	rs340808	C/G	IL5RA	3	3088831	No Data	intron[NM_175726.1]
542	rs340813	G/T	IL5RA	3	3099500	4	intron[NM_175726.1]
543	rs340833	A/G	IL5RA	3	3086458	5	utr-3[NM_175726.1]
544	rs34171309	G/T	CCL3	17	31440176	5	reference[NM_002983.2]
545	rs352140	C/T	TLR9	3	52231737	5	coding-synonymous[NM_017442.2]
546	rs353547	A/G	TWF2	3	52243906	1f	intron[NM_007284.3]
547	rs35797487	C/T	TJP2	9	71041824	No Data	missense[NM_201629.1];missense[NM_004817.2];reference[NM_004817.2]
548	rs369421	C/T	GATA3	10	8139183	2a	intron[NM_002051.2]
549	rs3733359	C/T	GC	4	72868638	5	utr-5[NM_000583.2]
550	rs3741240	A/G	SCGB1A1	11	61943118	No Data	utr-5[NM_003357.3]
551	rs3744246	C/T	ORMDL3	17	35337876	4	near-gene-5[NM_139280.1]
552	rs3744483	C/T	STAT3	17	37719964	4	utr-3[NM_213662.1];utr-3[NM_139276.2]
553	rs3746821	G/T	CD40	20	44188518	4	intron[NM_152854.2]
554	rs3747515	C/T	KCNS3	2	17977270	No Data	utr-3[NM_002252.3]
555	rs3747516	C/T	KCNS3	2	17976989	No Data	coding-synonymous[NM_002252.3]
556	rs3749985	C/G	HLA-DPB1	6	33162411	No Data	utr-3[NM_002121.4]
557	rs3752676	G/T	SPINK5	5	147430278	5	intron[NM_006846.2]
558	rs3755867	A/G	NFKB1	4	103745712	5	intron[NM_003998.2]
559	rs3756690	C/T	SPINK5	5	147487704	No Data	intron[NM_006846.2]
560	rs375947	A/G	IL12RB1	19	18041451	5	near-gene-3[NM_153701.1];reference[NM_005535.1]
561	rs3759635	C/T	STAT6	14	24048275	No Data	Promoter
562	rs3763349	C/T	PSMB8	6	32916210	5	near-gene-3[NM_148919.3]
563	rs3763354	C/T	HLA-DOB	6	32894895	6	-
564	rs3763364	A/T	PSMB8	6	32915498	5	near-gene-3[NM_148919.3]
565	rs3763365	C/T	PSMB8	6	32915431	5	near-gene-3[NM_148919.3]
566	rs376397	A/G	GATA3	10	8143304	4	intron[NM_002051.2]
567	rs3769683	A/G	CD28	2	204287042	2b	intron[NM_006139.1]
568	rs3769684	C/T	CD28	2	204293004	6	intron[NM_006139.1]
569	rs3774933	C/T	NFKB1	4	103645369	2a	intron[NM_003998.2]
570	rs3774938	A/G	NFKB1	4	103657469	6	intron[NM_003998.2]
571	rs3775073	A/G	TLR6	4	38506227	2b	reference[NM_006068.2]
572	rs3775152	G/T	GC	4	72849506	6	intron[NM_000583.2]
573	rs3777138	A/G	SPINK5	5	147475363	6	intron[NM_006846.2]
574	rs3777142	A/T	SPINK5	5	147467712	5	intron[NM_006846.2]
575	rs3780365	C/T	JAK2	9	5058520	No Data	intron[NM_004972.2]
576	rs3780372	C/T	JAK2	9	5087544	5	intron[NM_004972.2]
577	rs3780378	C/T	JAK2	9	5102288	No Data	intron[NM_004972.2]
578	rs3781093	A/G	GATA3	10	8141933	4	intron[NM_002051.2]
579	rs3782905	C/G	VDR	12	46552434	No Data	intron[NM_001017535.1]
580	rs3789873	C/G	TNC	9	116875035	6	intron[NM_002160.2]
581	rs3792421	C/T	IL5RA	3	3124791	5	intron[NM_175726.1]

582	rs3792424	C/T	IL5RA	3	3092700	No Data	intron[NM_175726.1]
583	rs3794766	C/T	NOS2A	17	23146048	1b	intron[NM_000625.3]
584	rs3799863	A/T	PLA2G7	6	46795750	No Data	intron[NM_005084.2]
585	rs3801266	A/G	NAMPT	7	105711486	4	intron[NM_005746.2]
586	rs3801267	A/T	NAMPT	7	105706158	No Data	intron[NM_005746.2]
587	rs3802600	A/T	GATA3	10	8148818	4	intron[NM_002051.2]
588	rs3803277	A/C	ALOX5AP	13	30216308	5	intron[NM_001629.2]
589	rs3804100	C/T	TLR2	4	154844859	No Data	coding-synonymous[NM_003264.3];
590	rs3804791	A/G	IL5RA	3	3124568	No Data	intron[NM_175727.1]
591	rs3804798	A/T	IL5RA	3	3102975	6	intron[NM_175726.1]
592	rs3804800	A/G	IL5RA	3	3102519	No Data	intron[NM_175726.1]
593	rs3804803	A/G	IL5RA	3	3099182	5	intron[NM_175726.1]
594	rs3806448	C/T	CHIA	1	111633728	5	near-gene-5[NM_201653.2]
595	rs3806933	C/T	TSLP	5	110434641	5	near-gene-5[NM_033035.3]
596	rs3815736	A/G	SPINK5	5	147473984	6	intron[NM_006846.2]
597	rs3818822	A/G	CHIA	1	111658731	No Data	missense[NM_201653.2];utr-5[NM_021797.2]
598	rs3819714	A/G	TAP2	6	32912195	6	intron[NM_018833.2]
599	rs3819721	A/G	TAP2	6	32912776	6	intron[NM_018833.2]
600	rs3824432	A/G	JAK2	9	5081675	No Data	intron[NM_004972.2]
601	rs3830058	C/T	HLA-DQB1	6	32741988	5	intron[NM_002123.3]
602	rs3846133	A/C	IL5RA	3	3103195	5	intron[NM_175726.1]
603	rs3856848	A/G	IL5RA	3	3122676	6	intron[NM_175726.1]
604	rs3859192	C/T	GSDM1	17	35382174	3a	intron[NM_178171.4]
605	rs3894194	C/T	GSDM1	17	35375519	1f	missense[NM_178171.4]
606	rs3917724	C/T	SELP	1	167848219	No Data	reference[NM_003005.3]
607	rs3917726	A/G	SELP	1	167847914	No Data	intron[NM_003005.3]
608	rs3917878	C/T	STAT6	17	29602968	No Data	Promoter
609	rs3918241	A/T	MMP9	20	44069142	5	near-gene-5[NM_004994.2]
610	rs3918392	A/G	ADAM33	20	3603219	5	reference[NM_153202.1];reference[NM_025220.2] ;missense[NM_025220.2]
611	rs3924264	A/G	NDUFS2	1	159445308	6	intron[NM_004550.4]
612	rs3939286	A/G	STAT6	9	6200099	6	Promoter
613	rs4001077	G/T	ACE	1	86702040	6	Promoter
614	rs404733	A/T	IL12RB1	19	18030997	5	near-gene-3[NM_005535.1]
615	rs4054760	C/T	IL5RA	3	3126668	3a	utr-5[NM_175728.1]
616	rs406103	C/T	GATA3	10	8151627	4	intron[NM_002051.2]
617	rs4072234	A/G	GUSB	1	238139849	No Data	Promoter
618	rs412681	A/G	GATA3	10	8154753	5	intron[NM_002051.2]
619	rs4143832	A/C	GSTP1	5	131890876	4	Promoter
620	rs4148871	C/T	TAP2	6	32911294	No Data	intron[NM_018833.2]
621	rs4148882	C/T	TAP1	6	32924936	6	intron[NM_000593.5]
622	rs4235232	G/T	TLR2	4	154837534	No Data	intron[NM_003264.3]
623	rs4239702	C/T	CD40	20	44182658	5	intron[NM_001250.4]

624	rs4240711	A/G	RXRA	9	136469127	5	utr-3[NM_002957.3]
625	rs425648	G/T	GSTP1	19	18063112	2b	Downstream
626	rs4270326	C/G	ICOS	2	204529859	No Data	intron[NM_012092.2]
627	rs4290	C/T	CLCA1	17	58905860	4	Promoter
628	rs4291	A/T	ACE	17	58907926	4	near-gene-5[NM_000789.2]
629	rs4295	C/G	ACE	17	58910030	4	intron[NM_000789.2]
630	rs4309	C/T	ACE	17	58913655	4	reference[NM_000789.2]
631	rs432014	C/T	IL1RN	2	113605050	6	intron[NM_173843.1];intron[NM_000577.3];intron[NM_173842.1]
632	rs4322988	G/T	IL5RA	3	3095571	No Data	intron[NM_175726.1]
633	rs4343	A/G	ACE	17	58919763	2b	coding-synonymous[NM_152830.1]
634	rs4362	C/T	ACE	17	58927493	5	coding-synonymous[NM_152830.1]
635	rs436857	A/G	IL12RB1	19	18058635	2b	utr-5[NM_153701.1]
636	rs4372063	A/G	JAK2	9	4993338	No Data	intron[NM_004972.2]
637	rs4393129	C/T	CLCA1	1	86702501	6	Promoter
638	rs444929	C/T	GATA3	10	8150030	No Data	intron[NM_002051.2]
639	rs4468448	C/T	ALOX5AP	13	30228316	No Data	intron[NM_001629.2]
640	rs4472254	G/T	SPINK5	5	147433830	6	intron[NM_006846.2]
641	rs4488202	A/G	SCGB1A1	11	61942174	5	near-gene-5[NM_003357.3]
642	rs4489574	C/T	CLCA1	1	159456337	1f	Promoter
643	rs4498841	A/C	JAK1	1	65191601	No Data	intron[NM_002227.2]
644	rs4516432	C/T	DPP10	2	116098367	No Data	intron[NM_001004360.2]
645	rs4529181	C/G	SPINK5	5	147449164	5	intron[NM_006846.2]
646	rs4559	A/G	NAB2	12	55775915	2b	near-gene-3[NM_005967.3]
647	rs4588	A/C	GC	4	72837187	5	missense[NM_000583.2]
648	rs4648006	C/T	NFKB1	4	103680588	No Data	intron[NM_003998.2]
649	rs4648064	A/G	NFKB1	4	103735775	6	intron[NM_003998.2]
650	rs4648090	A/G	NFKB1	4	103746106	6	intron[NM_003998.2]
651	rs4648093	A/G	NFKB1	4	103746693	5	coding-synonymous[NM_003998.2]
652	rs4648130	C/T	NFKB1	4	103755084	6	intron[NM_003998.2]
653	rs4648134	A/G	NFKB1	4	103755487	No Data	intron[NM_003998.2]
654	rs4648141	A/G	NFKB1	4	103755947	5	intron[NM_003998.2]
655	rs4656112	A/T	CLCA1	1	86702959	No Data	Promoter
656	rs4664448	A/G	GCG	2	162715666	No Data	intron[NM_002054.2]
657	rs4674259	A/G	IL8RB	2	218699250	1f	utr-5[NM_001557.2]
658	rs4675363	C/T	CD28	2	204298316	No Data	intron[NM_006139.1]
659	rs4696483	C/T	TLR2	4	154838705	5	intron[NM_003264.3]
660	rs4705916	A/C	CSF2	5	131435392	5	near-gene-5[NM_000758.2]
661	rs4715291	C/T	SERPINE1	6	52221319	No Data	Downstream
662	rs4720003	C/T	NOD1	7	30475517	4	intron[NM_006092.1]
663	rs4740	A/G	EBI3	19	4187996	No Data	missense[NM_005755.2]
664	rs477292	A/G	OPRM1	6	154405080	5	intron[NM_001008505.1]; intron[NM_000914.2]; intron[NM_001008503.1]

665	rs4787948	A/G	IL4R	16	27248560	1f	intron[NM_001008699.1]
666	rs4790210	A/G	SERPINE1	17	4475587	5	Downstream
667	rs4794067	C/T	TBX21	17	43163827	5	near-gene-5[NM_013351.1]
668	rs4795095	A/G	CCL5	17	31221413	6	near-gene-3[NM_002985.2]
669	rs4795402	A/C	MMP8	17	35338911	1f	Promoter
670	rs4796123	C/T	MMP8	17	31235573	6	Promoter
671	rs4796793	C/G	NAMPT	17	37795736	1f	Promoter
672	rs4832524	A/G	KCNS3	2	17977104	No Data	reference[NM_002252.3]
673	rs4833095	C/T	TLR1	4	38476105	5	reference[NM_003263.3]
674	rs4848306	A/G	LOC149620	2	113314578	6	Downstream
675	rs485411	C/T	FLJ45983	10	8133191	4	reference[NM_207423.1]
676	rs4857855	C/T	TEX12	3	129743240	No Data	Promoter
677	rs4880158	C/T	TRAF2	9	138924500	5	intron[NM_021138.3]
678	rs4916011	C/T	JAK1	1	65164614	No Data	intron[NM_002227.2]
679	rs4933	C/T	DAP3	1	153965742	4	coding- synonymous[NM_033657.1];coding-synonymous[NM_004632.2];reference[NM_004632.2]
680	rs4950928	C/G	CHI3L1	1	201422505	1f	utr-5[NM_001276.2]
681	rs4986832	A/G	ALOX5	10	45188034	5	near-gene-5[NM_000698.2]
682	rs502581	A/C	MS4A2	11	59616754	5	intron[NM_000139.2]
683	rs5030710	C/T	TLR4	9	119514542	5	coding-synonymous[NM_138554.2]
684	rs5030717	A/G	TLR4	9	119513655	No Data	intron[NM_138554.2]
685	rs5030729	A/G	TLR4	9	119514160	No Data	intron[NM_138554.2]
686	rs512555	A/G	MS4A2	11	59619829	6	utr-3[NM_000139.2]
687	rs521143	G/T	GATA3	10	8135781	4	near-gene-5[NM_207423.1];near-gene-5[NM_001002295.1]
688	rs5244	C/T	CMA1	14	24047434	4	near-gene-5[NM_001836.2]
689	rs5245	A/T	CMA1	14	24047341	4	near-gene-5[NM_001836.2]
690	rs5248	A/G	CMA1	14	24045654	No Data	intron[NM_001836.2]
691	rs525389	A/G	CHRM3	1	237999005	6	intron[NM_000740.2]
692	rs527004	C/T	CD80	3	120751802	5	intron[NM_005191.3]
693	rs528557	C/G	ADAM33	20	3599742	4	coding-synonymous[NM_153202.1]
694	rs528778	C/T	GATA3	10	8152149	3a	intron[NM_002051.2]
695	rs549908	G/T	IL18	11	111526126	No Data	reference[NM_001562.2]
696	rs556960	A/G	FLJ45983	10	8134152	4	intron[NM_207423.1]
697	rs569421	C/T	GATA3	10	8148598	5	intron[NM_002051.2]
698	rs570613	A/G	GATA3	10	8146508	5	intron[NM_002051.2]
699	rs5743270	A/G	NOD2	16	49299563	5	intron[NM_022162.1]
700	rs5743334	C/G	NOD1	7	30465636	4	intron[NM_006092.1]
701	rs5743356	G/T	NOD1	7	30452524	5	intron[NM_006092.1]
702	rs5743367	C/T	NOD1	7	30434903	6	intron[NM_006092.1]
703	rs5743369	A/G	NOD1	7	30434709	No Data	intron[NM_006092.1]
704	rs5743371	A/G	NOD1	7	30431899	No Data	intron[NM_006092.1]
705	rs5743808	C/T	TLR6	4	38507131	5	missense[NM_006068.2]

706	rs5743810	C/T	TLR6	4	38506745	No Data	missense[NM_006068.2]
707	rs5744295	A/C	IL1B	1	86704633	6	Promoter
708	rs5744455	C/T	CD14	5	139993491	3a	near-gene-5[NM_001040021.1]
709	rs5746065	A/C	TNFRSF1B	1	12190051	5	utr-3[NM_001066.2]
710	rs5746068	C/G	TNFRSF1B	1	12190142	5	utr-3[NM_001066.2]
711	rs5746847	C/T	TXNRD2	22	18301003	2b	intron[NM_006440.3]
712	rs5993883	G/T	COMT	22	18317638	No Data	intron[NM_000754.2]
713	rs6017737	C/T	IL1B	20	44176718	6	Promoter
714	rs6098	A/G	IL1B	18	59715374	6	reference[NM_002575.1]
715	rs6103	C/G	IL1B	18	59721483	1d	reference[NM_002575.1]
716	rs6104419	A/G	IL1B	20	44067296	4	Promoter
717	rs612020	C/T	IL1B	11	67102017	1f	Promoter
718	rs612242	C/G	IL17F	6	52211119	No Data	intron[NM_052872.3]
719	rs612709	A/G	ADAM33	20	3600207	3a	intron[NM_153202.1]
720	rs6131	A/G	SELP	1	167847509	6	missense[NM_003005.3]
721	rs6131034	C/G	CDH22	20	44257668	5	intron[NM_021248.1]
722	rs6133	G/T	SELP	1	167831970	4	reference[NM_003005.3]
723	rs614251	G/T	IL1RN	1	239872674	4	Promoter
724	rs624035	A/G	IL1RN	3	120739769	No Data	intron[NM_005191.3]
725	rs626364	C/T	CD80	3	120755573	5	intron[NM_005191.3]
726	rs6413523	G/T	SFTPD	10	81696115	6	intron[NM_003019.4]
727	rs6425925	C/G	IL1RN	1	35146797	5	Downstream
728	rs6476934	G/T	JAK2	9	4983267	5	intron[NM_004972.2]
729	rs6476939	A/T	JAK2	9	5074837	5	intron[NM_004972.2]
730	rs6498011	A/G	IL4R	16	27239395	No Data	intron[NM_001008699.1]
731	rs6500328	A/G	NOD2	16	49294157	5	intron[NM_022162.1]
732	rs6580521	A/T	SPINK5	5	147454992	6	intron[NM_006846.2]
733	rs6587665	C/T	LOC100128	1	150557595	No Data	intron[XM_001718271.1]
734	rs6590986	C/T	MMP8	11	102104069	2a	-
735	rs6627	A/G	MAML1	5	179136539	5	utr-3[NM_014757.3]
736	rs6665683	C/T	FCER1A	1	157531752	No Data	intron[NM_002001.2]
737	rs6666554	A/G	SELP	1	167868733	No Data	-
738	rs6672024	C/T	FCER1A	1	157533346	5	intron[NM_002001.2]
739	rs6680548	C/T	JAK1	1	65187031	5	intron[NM_002227.2]
740	rs6691378	A/G	ARG1	1	201423745	6	Promoter
741	rs6707930	A/C	IL1F9	2	113454101	No Data	missense[NM_019618.2]
742	rs6735355	A/G	DPP10	2	116264996	6	intron[NM_020868.2]
743	rs677901	C/T	ARG1	6	52219060	No Data	Promoter
744	rs6793085	C/T	IL5RA	3	3117423	5	intron[NM_175728.1]
745	rs6807043	C/T	CD80	3	120747877	6	intron[NM_005191.3]
746	rs6807532	C/T	CD80	3	120757531	5	intron[NM_005191.3]
747	rs6808378	C/T	IL5RA	3	3117943	No Data	intron[NM_175728.1]

748	rs6809408	A/C	IL5RA	3	3097827	No Data	intron[NM_175726.1]
749	rs6810204	C/T	CD80	3	120757788	5	intron[NM_005191.3]
750	rs68600	A/G	HLA-DMB	6	33011702	5	intron[NM_002118.3]
751	rs6864123	A/G	TSLP	5	110441032	No Data	utr-3[NM_033035.3]
752	rs6864920	A/G	SPINK5	5	147454820	5	intron[NM_006846.2]
753	rs6872218	C/T	SPINK5	5	147486183	No Data	intron[NM_006846.2]
754	rs6872265	G/T	SPINK5	5	147473736	No Data	intron[NM_006846.2]
755	rs6872979	C/G	SPINK5	5	147444035	No Data	intron[NM_006846.2]
756	rs6878956	C/T	SPINK5	5	147474552	No Data	intron[NM_006846.2]
757	rs688659	A/G	OPN3	1	239868218	4	intron[NM_014322.2]
758	rs6892070	C/T	SPINK5	5	147494433	No Data	intron[NM_006846.2]
759	rs6892205	A/G	SPINK5	5	147455579	No Data	reference[NM_006846.2]
760	rs689470	C/T	PTGS2	1	184907681	5	utr-3[NM_000963.1]
761	rs6896451	A/G	SPINK5	5	147495354	No Data	intron[NM_006846.2]
762	rs6905503	G/T	TAP2	6	32902097	5	intron[NM_018833.2]
763	rs6906021	C/T	HLA-DQB1	6	32734289	1d	near-gene-3[NM_002123.3]
764	rs6912414	A/G	HLA-DOB	6	32882443	No Data	-
765	rs693640	C/T	CD80	3	120747072	No Data	intron[NM_005191.3]
766	rs6949758	C/G	NOD1	7	30468204	No Data	intron[NM_006092.1]
767	rs6958905	C/T	NPSR1	7	34856077	No Data	intron[NM_207173.1]
768	rs6959470	C/T	NOD1	7	30436415	5	intron[NM_006092.1]
769	rs6963954	A/G	NOD1	7	30480966	No Data	intron[NM_006092.1]
770	rs6972158	A/G	NPSR1	7	34855707	6	intron[NM_207173.1]; missense[NM_207172.1]
771	rs7030260	A/C	JAK2	9	4998070	No Data	intron[NM_004972.2]
772	rs7031456	G/T	JAK2	9	5069156	No Data	intron[NM_004972.2]
773	rs7046736	A/C	JAK2	9	5005732	No Data	intron[NM_004972.2]
774	rs705119	A/C	GC	4	72831900	No Data	intron[NM_000583.2]
775	rs705124	A/G	GC	4	72851507	No Data	intron[NM_000583.2]
776	rs709607	C/G	GUSB	7	65086976	4	-
777	rs7096206	C/G	MBL2	10	54201691	1f	near-gene-5[NM_000242.1]
778	rs711438	A/G	NAMPT	7	105694547	No Data	intron[NM_005746.2]
779	rs714158	C/T	CYFIP2 ;	5	156631883	4	intron[NM_001037333.1];intron[NM_001037332.2]
780	rs714289	C/T	HLA-DMB	6	33013789	6	intron[NM_002118.3]
781	rs7179270	C/T	TJP1	15	27805919		reference[NM_175610.2];reference[NM_003257.3];missense[NM_003257.3]
782	rs7196646	A/G	NLRC3	16	3560288	6	intron[NM_178844.2]
783	rs7216389	C/T	GSDML	17	35323475	6	intron[NM_018530.2]
784	rs7217186	C/T	ALOX15	17	4486141	5	intron[NM_001140.3]
785	rs7219923	C/T	GSDML	17	35328044	6	intron[NM_001042471.1]
786	rs722323	C/T	IL17F	6	52214819	No Data	intron[NM_052872.3]
787	rs7224129	A/G	ORMDL3	17	35328952	1f	near-gene-3[NM_139280.1]
788	rs7246264	C/T	FCER2	19	7668978	5	intron[NM_002002.3]
789	rs726168	C/T	CHRM3	1	237860987	3b	intron[NM_000740.2]

790	rs7269320	C/T	VISA	20	3794397	5	reference[NM_020746.2]
791	rs727162	C/G	NPSR1	7	34840563	6	reference[NM_207173.1];reference[NM_207172.1];missense[NM_207172.1]
792	rs727588	A/C	CYFIP2	5	156631707	2a	intron[NM_001037333.1];intron[NM_001037332.2]
793	rs736782	C/T	NOD1	7	30470798	3a	intron[NM_006092.1]
794	rs737866	A/G	COMT	22	18310109	4	intron[NM_000754.2]
795	rs7383287	A/G	HLA-DOB	6	32891064	5	coding-synonymous[NM_002120.3]
796	rs7419168	C/T	JAK1	1	65169610	No Data	intron[NM_002227.2]
797	rs7502875	A/C	TBX21	17	43178226	5	utr-3[NM_013351.1]
798	rs7515776	A/T	CHI3L1	1	201422326	1f	intron[NM_001276.2]
799	rs7523783	A/G	JAK1	1	65145205	4	intron[NM_002227.2]
800	rs7524842	C/T	JAK1	1	65103379	No Data	intron[NM_002227.2]
801	rs7528403	G/T	JAK1	1	65155380	5	intron[NM_002227.2]
802	rs7539178	A/C	JAK1	1	65155590	2b	intron[NM_002227.2]
803	rs7542760	A/G	PIGR	1	205174429	No Data	reference[NM_002644.2]
804	rs7553101	C/T	JAK1	1	65192778	No Data	intron[NM_002227.2]
805	rs7569719	C/T	KCNS3	2	17975939	No Data	coding-synonymous[NM_002252.3]
806	rs757716	C/G	CD14	7	100574122	1f	Downstream
807	rs761988	A/G	CD14	14	24050525	6	Downstream
808	rs7628626	A/C	CD80	3	120727111	No Data	utr-3[NM_005191.3]
809	rs763780	C/T	IL17F	6	52209698	No Data	reference[NM_052872.3]
810	rs7639988	C/T	IL5RA	3	3108584	No Data	intron[NM_175726.1]
811	rs7647903	A/G	IL5RA	3	3108791	No Data	utr-3[NM_175727.1]
812	rs7653908	C/G	TLR10	4	38458616	6	intron[NM_030956.2]
813	rs7658893	A/G	TLR10	4	38458815	No Data	intron[NM_030956.2]
814	rs767007	C/G	CYFIP2	5	156629073	4	intron[NM_001037333.1];intron[NM_001037332.2]
815	rs7674579	G/T	TLR2	4	154834184	No Data	intron[NM_003264.3]
816	rs769178	A/C	LTB	6	31655493	2b	near-gene-3[NM_009588.1]
817	rs7711953	C/G	SPINK5	5	147448427	No Data	intron[NM_006846.2]
818	rs7748681	A/C	HLA-DOB	6	32881836	No Data	-
819	rs7762120	C/T	HLA-DOB	6	32893262	6	near-gene-5[NM_002120.3]
820	rs7781700	C/T	NOD1	7	30464057	No Data	intron[NM_006092.1]
821	rs778583	C/T	ADRB2	5	139985294	No Data	Downstream
822	rs778584	C/T	FCER1G	5	139985396	1f	Downstream
823	rs778593	C/T	IK	5	140007400	4	near-gene-5[NM_006083.3]
824	rs7789066	A/G	SELP	7	105714988	No Data	Promoter
825	rs7793010	C/T	NOD1	7	30436678	4	intron[NM_006092.1]
826	rs7794325	C/G	NOD1	7	30433453	No Data	intron[NM_006092.1]
827	rs7849191	C/T	JAK2	9	4978761	No Data	intron[NM_004972.2]
828	rs7851969	C/G	JAK2	9	5090291	No Data	intron[NM_004972.2]
829	rs7852309	A/G	JAK2	9	5000920	6	intron[NM_004972.2]
830	rs7852970	A/G	TRAF2	9	138940242	2b	utr-3[NM_021138.3]
831	rs7854413	C/T	PDCD1LG2	9	5547708	5	missense[NM_025239.2]

832	rs7857730	G/T	JAK2	9	5074049	No Data	intron[NM_004972.2]
833	rs7859390	A/T	JAK2	9	5052473	6	intron[NM_004972.2]
834	rs7864330	G/T	TLR4	9	119509371	6	intron[NM_138554.2]
835	rs7865082	A/G	DDX58	9	32447558	6	intron[NM_014314.3]
836	rs7865462	C/T	TNC	9	116862935	No Data	intron[NM_002160.2]
837	rs7869402	C/T	TLR4	9	119517853	4	utr-3[NM_138554.2]
838	rs7897947	G/T	NFKB2	10	104147701	2a	intron[NM_002502.3];intron[NM_001077493.1]
839	rs7943404	A/G	CH13L1	11	102103785	No Data	Promoter
840	rs7970260	A/C	ICOS	12	6419322	6	Promoter
841	rs8057341	A/G	NOD2	16	49295481	6	intron[NM_022162.1]
842	rs8075442	C/T	STAT3	17	37736929	No Data	intron[NM_213662.1];intron[NM_139276.2]
843	rs8078731	A/T	STAT3	17	37733907	6	intron[NM_213662.1];intron[NM_139276.2]
844	rs8079416	C/T	IL10	17	35346239	6	Promoter
845	rs8140265	A/G	TXNRD2	22	18301641	No Data	intron[NM_006440.3]
846	rs816293	C/G	NOS1	12	116247082	6	intron[NM_000620.1]
847	rs8176348	C/T	CYP27B1	12	56443400	5	intron[NM_000785.3]
848	rs8176353	A/T	CYP27B1	12	56441489	5	near-gene-3[NM_000785.3]
849	rs843429	A/G	DPP10	2	115937478	No Data	intron[NM_001004360.2]
850	rs867174	A/G	JAK3	19	17813930	4	intron[NM_000215.2]
851	rs8674	C/T	IL4R	16	27283390	2b	utr-3[NM_000418.2]
852	rs869402	C/T	GSDML	17	35321569	6	intron[NM_018530.2]
853	rs880633	C/T	CHI3L1	1	201419424	5	reference[NM_001276.2]
854	rs880678	A/G	CMA1	14	24044884	6	intron[NM_001836.2]
855	rs9034	C/T	NAMPT	7	105677292	5	utr-3[NM_005746.2]
856	rs906134	C/G	GUSB	7	65081723	No Data	intron[NM_000181.2]
857	rs913594	C/T	JAK2	9	5053199	No Data	intron[NM_004972.2]
858	rs916055	C/T	ALOX15	17	4481583	No Data	utr-3[NM_001140.3]
859	rs9270665	G/T	HLA-DRB1	6	32674210	6	-
860	rs9277378	A/G	HLA-DPB1	6	33158257	6	intron[NM_002121.4]
861	rs9277419	A/G	HLA-DPB1	6	33159755	No Data	intron[NM_002121.4]
862	rs9277535	A/G	HLA-DPB1	6	33162839	6	utr-3[NM_002121.4]
863	rs9277935	G/T	RXRB	6	33268403	1b	near-gene-5[NM_080680.2];near-gene-5[NM_080679.2];near-gene-3[NM_021976.3]
864	rs9282745	A/T	IL4	5	132041899	5	intron[NM_172348.1]
865	rs9303277	C/T	IKZF3	17	35229995	6	intron[NM_183232.1]
866	rs93059	C/T	NFKB1	4	103687547	1f	intron[NM_003998.2]
867	rs932272	C/T	NOD1	7	30479684	3a	intron[NM_006092.1]
868	rs933271	C/T	COMT	22	18311407	4	intron[NM_000754.2]
869	rs944724	C/T	NOS2A	17	23133544	5	intron[NM_000625.3]
870	rs944725	C/T	NOS2A	17	23133698	No Data	intron[NM_000625.3]
871	rs9462851	A/G	PTCRA	6	42998580	6	intron[NM_138296.2]
872	rs9471960	A/C	PTCRA	6	42992728	No Data	intron[NM_138296.2]
873	rs9494145	C/T	MYB	6	135474245	No Data	-

874	rs9500928	C/T	HLA-DPB1	6	33157672	6	intron[NM_002121.4]
875	rs9509	C/T	MMP9	20	44078560	6	utr-3[NM_004994.2]
876	rs9530	C/T	GUSB	7	65063329	5	missense[NM_000181.2]
877	rs9658562	A/T	NOS1	12	116136440	6	utr-3[NM_000620.1]
878	rs966871	A/T	JAK2	9	5111070	No Data	intron[NM_004972.2]
879	rs9729	A/C/G	VDR	12	46522890	5	utr-3[NM_000376.2]
880	rs9784858	C/G	HLA-DOB	6	32895153	6	-
881	rs9784876	A/C	TAP2	6	32896856	5	near-gene-3[NM_018833.2]
882	rs9836399	A/G	CD86	3	123294501	6	intron[NM_006889.3]
883	rs9848900	A/G	CD86	3	123314187	6	intron[NM_006889.3]
884	rs9855093	A/G	CD80	3	120731011	6	intron[NM_005191.3]
885	rs9869655	A/G	IL5RA	3	3096857	6	intron[NM_175726.1]
886	rs9883988	A/G	COL29A1	3	131645085	6	reference[NM_153264.4]
887	rs988885	C/T	SPINK5	5	147468178	No Data	intron[NM_006846.2]
888	rs9890802	A/C	STAT3	17	37747327	5	intron[NM_213662.1]; intron[NM_139276.2]
889	rs9901146	A/G	CHRM3	17	35296869	6	Downstream
890	rs9912773	C/G	STAT3	17	37764060	1f	intron[NM_213662.1]; intron[NM_139276.2]
891	rs9926974	C/T	NLRC3	16	3565568	5	intron[NM_178844.2]
892	rs9930893	A/C	NLRC3	16	3565449	5	intron[NM_178844.2]
893	rs993986	C/T	DPP10	2	115819691	No Data	intron[NM_020868.2]
894	rs9972960	A/G	CHML	17	31444192	5	Promoter
895	rs9986640	A/G	HLA-DOB	6	32872948	2a	-

Alleles: An alternative form of a gene (one member of a pair) that is located at a specific position on a specific chromosome.
Gene: Selected Genes
Chr: Chromosome number stands for gene position
Position: Chromosome Position stands for SNP position on gene.
Regulome DB score: This is the scoring scheme refers to the following available data types for a single coordinate: 1a: eQTL + TF binding + matched TF motif + matched DNase Footprint + DNase peak; 1b: eQTL + TF binding + any motif + DNase Footprint + DNase peak; 1c: eQTL + TF binding + matched TF motif + DNase peak; 1d: eQTL + TF binding + any motif + DNase peak; 1f: eQTL + TF binding / DNase peak; 2a: TF binding + matched TF motif + matched DNase Footprint + DNase peak; 2b: TF binding + matched TF motif + matched DNase Footprint + DNase peak; 2c: TF binding + matched TF motif + DNase peak; 3a: TF binding + any motif + DNase peak; 3b: TF binding + matched TF motif; 4: TF binding + DNase peak; 5: TF binding or DNase peak; and 6: others.

Table S2. Clinical information

ID	Race	Gender	Age at IgE testing	Total IgE	d1	d2	I6	e1	e5	m6
**1**	Hispanic	male	6.7707	36.99	0.183	0	0.026	0.007	0.0358	0.0238
**2**	Black / African American	female	8.62149	643.36	0.034	0.018	0.045	0.018	0.0405	0.1116
**3**	Black / African American	female	8.03559	29.93	0.01	0	0.012	0.007	0.0458	0.0329
**4**	Hispanic	male	6.92129	1869.35	0.748	0	0.56	126.819	66.1872	2.2295
**5**	Hispanic	female	7.21971	755.75	1.451	0	83.25	0.04	0.1094	0.0493
**6**	Black / African American	male	6.68309	44.42	0.016	0	0.012	0.002	0.0173	0.2193
**7**	Hispanic	male	6.51061	8.34	0.055	0	0.018	0	0.0114	0.0399
**8**	Black / African American	male	8.50924	49.68	0.018	0	0.004	0.004	0.0122	0.0757
**9**	Black / African American	male	7.47707	9.41	0.005	0	0	0	0.0129	0.0268
**10**	Black / African American	female	8.61602	2101.83	0.812	0.7698	1.864	7.405	2.438	54.6995
**11**	Hispanic	female	7.59206	189.96	6.924	0	0.054	0.009	0.0733	0.0475
**12**	Black / African American	female	7.50719	60.57	0.899	0	0.089	0	0.0245	0.1265
**13**	White	female	7.99452	26.78	0.004	0	0.007	0	0.0068	0.0958
**14**	Hispanic	male	6.57632	55.53	2.686	0	0.131	2.967	0.4683	0.0328
**15**	Cap Verdean	male	6.57084	14.18	0	0	0	0	0.0049	0.0396
**16**	Black / African American	male	8.36961	125.89	13.905	3.8052	0.017	0.027	0.0533	0.108
**17**	Hispanic	male	6.73238	28.89	0.264	0	1.727	0.056	0.01	0.0442
**18**	Black / African American	male	8.70363	37.19	0.029	0.016	0.165	0.006	0.0658	0.0613
**19**	Black / African American	male	7.16769	400.21	0.011	0	0.006	0.02	0.0449	0.0275
**20**	Black / African American	female	8.01643	86.68	0.265	0.3021	0.062	0.023	0.0491	0.0904
**21**	Hispanic	male	7.60849	44.75	0.003	0	0.004	0.001	0.0157	0.042
**22**	Black / African American	male	7.07187	50.95	0	0	0	1.245	0.0895	0.0173
**23**	Black / African American	female	5.99589	68.82	0.012	0	0	0.748	21.6354	0.0248
**24**	White	male	6.81177	2.42	0.004	0	0.003	0	0.0065	0.0764
**25**	Black / African American	female	7.65229	142.5	0.012	0.016	0.049	0.027	0.1143	0.154
**26**	Black / African American	female	6.04791	166.76	0.021	0	0.01	0.006	0.0215	0.0243
**27**	Black / African American	male	6.84736	19.59	0	0	0	0	0.0036	0.0371
**28**	Black / African American	female	5.86721	7.52	0	0	0	0.548	0.0423	0.2703
**29**	Unknown	female	6.02601	54.22	0.066	0	0	0.034	0.0297	0.0395
**30**	mixed	male	5.295	238.78	0.035	0	0.028	2.062	0.1383	0.064
**31**	Black / African American	female	6.02875	172.27	0.013	0	0.681	0.008	0.0173	0.0206
**32**	Black / African American	female	6.27515	1007.21	1.261	0	0.841	14.874	6.0565	5.9859
**33**	Black / African American	male	6.55441	63.75	0	0	0	0	0.0006	0.0275
**34**	Black / African American	female	7.02806	1.76	0.006	0	0.012	0.003	0.0137	0.067
**35**	Unknown	male	8.47912	1513.05	18.805	25.6269	0.195	4.15	3.4695	17.1599
**36**	Black / African American	female	6.73238	537.36	0.11	0.0821	0.061	3.532	1.4427	0.1147
**37**	Black / African American	female	5.32786	76.15	0	0	0	0	0.007	0.0127

**38**	Hispanic	male	6.91034	45.39	0.628	0	0.138	0.007	0.0615	0.0195
**39**	Ascian/ Pacific	male	6.59001	30.94	0	0	0	0.009	0.0166	0.0136
**40**	Black / African American	female	7.50719	81.98	0.008	0.0067	0.007	0.01	0.0035	0.0721
**41**	Black / African American	male	5.577	27.66	0.219	0	0.031	0	0.0138	0.0231
**42**	Black / African American	female	4.98563	853.39	0.171	0	3.738	10.546	0.3074	0.0422
**43**	Black / African American	male	5.8371	8.76	0.006	0	0.006	0	0.0037	0.0187
**44**	Black / African American	female	5.09514	43.84	0.139	0	0.022	0	0.0158	0.0766
**45**	Hispanic	male	6.52704	39.63	0.001	0	0.033	0	0.0591	0.0279
**46**	Black / African American	male	6.00411	27.42	0.001	0	0.001	0.008	0.0035	0.0472
**47**	Black / African American	male	8.21903	37.31	0.037	0.0246	0.015	0.002	0.0078	0.0679
**48**	Black / African American	male	4.50376	81.99	0.35	0	0.124	0	0.0809	0.0605
**49**	Black / African American	female	8.20808	43.98	4.953	5.4417	0.02	0.401	0.0999	0.2862
**50**	Cap Verdean	female	6.93224	3.82	0.017	0	0.003	0	0.0073	0.0296
**51**	Black / African American	male	6.59001	87.08	0.009	0	0.011	0.014	0.0251	0.0275
**52**	Black / African American	female	5.11978	81.44	0.019	0	0.016	0.004	0.0147	0.0224
**53**	Cap Verdean	male	5.24572	30.24	0.008	0	0.013	0.002	0.0121	0.0342
**54**	Hispanic	female	6.11636	185.54	0	0	0.852	0.777	0.0838	0.0179
**55**	Hispanic	female	4.75838	213.12	0.092	0	0.005	0.004	0.0201	0.0133
**56**	Black / African American	female	3.93429	2603.72	1.386	0	5.132	60.248	8.3687	3.7929
**57**	Black / African American	female	5.82067	104.88	0.018	0.0063	0.043	0	0.0074	0.1099
**58**	Unknown	female	7.09925	29.69	0.004	0	0	0.002	0.0278	0.107
**59**	Black / African American	male	4.34223	33.67	0.008	0	0.152	0.001	0.001	0.0237
**60**	Hispanic	male	6.14648	148.7	1.517	0	2.817	0.217	0.7459	0.0425
**61**	Black / African American	female	7.04997	24.16	0.007	0	0.009	0	0.0116	0.0714
**62**	Hispanic	female	5.01574	15.98	2.98	0	0.782	0.04	0.1887	0.0365
**63**	Hispanic	female	6.93771	9.64	0.008	0.0011	0.002	0.007	0.0173	0.0684
**64**	Black / African American	female	4.21629	11.66	0	0	0	0	0.0137	0.0197
**65**	Black / African American	male	3.89596	21.21	0.016	0	0.01	0	0.0063	0.0204
**66**	Black / African American	female	6.66119	42.7	0.005	0	0.004	0.001	0.0075	0.0632
**67**	White	male	5.09788	0.43	0.14	0.0215	0	0.001	0.0061	0.0873
**68**	Black / African American	male	4.01369	6.61	0	0	0	0	0.0071	0.0224
**69**	Cap Verdean	female	5.40726	76.25	10.568	0	0	0	0.023	0.0345
**70**	Black / African American	female	4.03012	57.23	0	0	0	0	0.0147	0.0207
**71**	Black / African American	male	6.06708	89.81	1.628	0	0.092	0.004	0.0494	0.0095
**72**	Black / African American	female	3.53457	103.7	0.003	0	0.001	0	0.0173	0.0217
**73**	Black / African American	male	3.64408	73.22	1.283	0	2.694	0.021	0.0881	0.0367
**74**	Black / African American	male	4.36413	246.37	68.644	0	0.013	0.034	0.1832	0.0333
**75**	Black / African American	female	5.88912	1419.93	1.749	0	95.517	1.673	13.8196	0.3017
**76**	White	female	6.57084	403.05	0.066	1.6803	0.033	0.858	1.9411	0.1012
**77**	Cap Verdean	female	5.06229	580.8	57.595	0	0.006	0.019	0.0845	0.0281
**78**	Black / African American	male	3.52088	1426.93	158.919	0	0.092	0.094	0.4589	0.0603
**79**	Black / African American	female	6.59274	53.2	0.006	0	0.005	0.007	0.0057	0.0904
**80**	Hispanic	female	5.10883	785.85	0.215	0.207	0.151	133.1	20.8546	0.2253
**81**	Black / African American	male	5.68378	28.46	0	0	0	0	0.0119	0.0127

**82**	Black / African American	female	4.59138	63.36	0.804	0.179	0.003	0.005	0.0084	0.1032
**83**	Black / African American	female	3.19507	29.36	1.989	0	0.027	0.003	0.089	0.0002
**84**	Cap Verdean	female	4.8898	47.48	0.002	0	0	0.001	0.0294	0.0357
**85**	Hispanic	male	5.05955	29.99	0.028	0.0103	0.014	0.004	0.0132	0.0887
**86**	Hispanic	female	3.00616	120.89	0	0	0.105	1.971	0.1035	0.0241
**87**	Black / African American	male	4.25188	102.91	0.571	0	0.16	0.031	0.0514	0.0449
**88**	Black / African American	male	7.39493	28.82	0.065	0.0436	0.781	0	0.0173	0.0734
**89**	Black / African American	male	4.17522	103.19	0.434	0	0.072	0.005	0.0528	0.0504
**90**	Black / African American	male	3.27721	83.63	0	0	0	0	0.0217	0.0249
**91**	Hispanic	female	2.97878	121.51	0.343	0	0.199	0.06	0.092	0.0682
**92**	Black / African American	male	4.88159	32.32	0	0	0	0	0.0838	0
**93**	Hispanic	male	3.36482	27.41	0.011	0	0	0	0.0097	0.0448
**94**	Black / African American	male	3.49897	5.91	0	0	0	0	0.0061	0.0047
**95**	Black / African American	female	4.22998	88.27	24.159	25.3792	0.009	0.01	0.0209	0.0682
**96**	Ascian/ Pacific	female	3.00616	30.46	0	0	0	0.015	0.005	0.0409
**97**	Black / African American	female	5.56057	94.38	0.012	0	0	0	0.0109	0.0046
**98**	Black / African American	male	5.95209	54.21	0.008	0	0.01	0	0.0143	0.0219
**99**	Black / African American	male	3.50719	172.38	0.01	0	0.003	0.005	0.0297	0.0489
**100**	Hispanic	male	3.01985	117.4	0.007	0	0	0	0.0273	0.0065
**101**	Black / African American	male	2.46954	841.79	0.2	0	0.056	9.844	5.1819	0.0999
**102**	Black / African American	male	6.53799	13.49	0.012	0	0	0.002	0.0073	0.0701
**103**	Black / African American	male	5.11157	382.49	0.107	0	47.594	45.102	1.8382	0.0512
**104**	Hispanic	female	4.67899	362.24	0.075	0.0392	0.041	0.258	0.5012	0.1648
**105**	Black / African American	female	4.09856	20.88	0.007	0	0.007	0.001	0.007	0.0691
**106**	Black / African American	male	3.00616	11.86	0	0	0	0	0.0058	0.0488
**107**	Black / African American	male	6.62012	65.65	0.01	0	0.017	0.006	0.0404	0.1231
**108**	Hispanic	male	5.74675	53.94	0.012	0	0.02	0	0.019	0.0443
**109**	Hispanic	female	2.25325	11.13	0.005	0	0.001	0	0.0043	0.0879
**110**	Hispanic	male	4.52841	36.78	0	0	0	0.001	0.0182	0.0358
**111**	Cap Verdean	male	4.59411	22	0.006	0	0.002	0	0.017	0.0092
**112**	Black / African American	female	4.11499	2.33	0.009	0	0.008	0	0.0103	0.1059
**113**	Hispanic	male	6.40931	23.67	0.022	0.0023	0.014	0.009	0.0124	0.087
**114**	Hispanic	male	4.92539	154.78	0	0	0	0.008	0.065	0.0297
**115**	Black / African American	female	4.5859	18.85	0	0	0.026	0	0.0545	0
**116**	Black / African American	female	3.03901	11.13	0.05	0	0.008	0	0.0058	0.0237
**117**	Black / African American	male	4.2026	13.69	0	0	0	0	0.008	0.0235
**118**	Hispanic	male	1.94387	65.35	0	0	0.004	0	0.0056	0.5695
**119**	Ascian/ Pacific	male	2.30527	15.46	0	0	0	0	0.0138	0.0195
**120**	Black / African American	male	6.05339	413.7	0.058	0.0387	0.045	6.238	0.6095	0.0788
**121**	Black / African American	female	2.53525	42.99	0.018	0	0.012	0.651	0.3607	0.0245
**122**	Cap Verdean	male	5.23477	28.3	0.009	0	0.011	0.007	0.0124	0.0181
**123**	Black / African American	male	1.7358	43.52	0.009	0	0.012	0.003	0.0387	0.2783
**124**	Hispanic	male	3.07461	87.14	0.831	0	0.13	0.025	0.0403	0.0501
**125**	Black / African American	male	2.72142	6.93	0.008	0	0.011	0	0.0327	0.0424

**126**	Hispanic	female	4.88433	329.95	0.463	0.2547	13.385	0.063	0.0944	0.0777
**127**	Black / African American	female	4.92539	42.84	0	0	0	0	0.008	0
**128**	Black / African American	female	4.01643	15.41	0.002	0	0.006	0	0.0075	0.0642
**129**	Black / African American	male	4.23272	112.09	0.029	0.0119	0.052	0.008	0.0698	0.0893
**130**	White	female	2.65845	13.27	0.004	0	0.011	0.001	0.0106	0.0479
**131**	Black / African American	female	2.49966	340.01	0.016	0	0.009	0.019	0.0435	0.0577
**132**	Black / African American	female	6.37372	116.07	0.109	0.0974	0.023	0	0.0318	0.403
**133**	Black / African American	male	5.28679	73.94	0.013	0	4.398	0.053	0.0269	0.0293
**134**	Black / African American	female	4.04654	10.45	0.01	0	0.005	0	0.0216	0.0195
**135**	Black / African American	female	3.2553	75	0.012	0.0016	0.008	0.003	0.0145	0.0664
**136**	Black / African American	female	2.15743	137.08	0.005	0	0.003	0.009	0.084	0.0066
**137**	Black / African American	female	2.00684	36.51	0	0	0.001	0	0.0019	0.0022
**138**	Black / African American	male	3.73169	35.59	2.183	3.4041	0.018	0.004	0.0301	0.1074
**139**	Unknown	male	2.04791	37.07	0.092	0	0.013	0	0.0144	0
**140**	Black / African American	male	5.02122	134.72	0.012	0	0.011	0.019	0.0242	0.022
**141**	Black / African American	male	2.87474	0	0.009	0	0.001	0.046	0.004	0.0729
**142**	Black / African American	male	3.54825	99.48	0.042	0.0151	0.01	0.002	0.0149	0.0952
**143**	Hispanic	female	5.20739	221.6	53.622	0	0.066	0.115	0.0827	0.0376
**144**	Black / African American	male	1.88638	75.1	0.503	0	0.112	0	0.0252	0.0205
**145**	Black / African American	female	6.19576	206.73	0.029	0.0115	0.023	0.018	0.0263	0.0851
**146**	Black / African American	female	1.67283	22.04	0	0	0	0.006	0.006	0.0413
**147**	Unknown	male	4.04381	32.87	0.006	0	0.01	0.004	0.0175	0.0215
**148**	Hispanic	male	4.08487	27.87	0	0	0	0	0.0112	0.017
**149**	Black / African American	female	4.16701	226.54	0.117	0	0	0.007	0.0319	0.0242
**150**	White	male	4.06845	620.3	105.671	0	1.098	0.566	0.2247	0.0175
**151**	Black / African American	female	1.6345	0.22	0	0	0	0	0.0035	0.02
**152**	Hispanic	male	2.88569	9.16	0.007	0	0.001	0.002	0.0035	0.069
**153**	Black / African American	female	1.99863	8.54	0.054	0.0381	0.013	0.004	0.0128	0.0912
**154**	Black / African American	male	4.03559	5.81	0.185	0	0.014	0	0.0027	0.0632
**155**	Black / African American	male	1.33333	537.22	12.982	0	17.436	0.182	0.2595	0.0452
**156**	Cap Verdean	female	1.01574	76.39	0.01	0	0.005	0.003	0.0549	0.0411
**157**	Hispanic	male	6.34086	122.2	0.046	0.014	0.024	0.044	0.3235	0.0702
**158**	Hispanic	female	1.05407	35.58	0.05	0	0.025	0	0.0444	0.0255
**159**	White	male	4.03285	82.81	0.012	0	0.007	0	0.0045	0.0255
**160**	Black / African American	male	0.83778	0.31	0	0	0	0	0.0079	0.0406
**161**	Hispanic	male	3.90418	73.7	0	0	0.007	0	0.0097	0.0173
**162**	Hispanic	male	1.00479	32.23	0	0	0	0	0.031	0.0443
**163**	White	male	1.57426	12.86	0.006	0	0.003	0.007	0.0031	0.097
**164**	Black / African American	female	1.46201	1039.86	16.814	0	32.182	2.175	1.2218	0.1218
**165**	Hispanic	male	0.83778	16.2	0.001	0	0.007	0	0.005	0.0267
**166**	Hispanic	female	1.04586	0	0	0	0	0	0.0016	0.0275
**167**	Black / African American	male	0.73101	18.59	0	0	0	0.029	0.0103	0.0221
**168**	Hispanic	female	1.99589	23.82	0.015	0	0.018	0.001	0.02	0.052
**169**	Black / African American	male	0.8104	24.4	0	0	0	0	0.0225	0

**170**	Black / African American	male	4.98289	342.95	0.213	0	0.055	0.007	0.0121	0.0362
**171**	Hispanic	male	5.06502	6.01	0.261	0	0.003	0	0.0155	0.0268
**172**	Hispanic	male	0.77207	4.14	0.002	0	0.018	0.021	0.0383	0.0199
**173**	Black / African American	male	0.67625	11.78	0	0	0	0	0.0073	0.0274
**174**	Black / African American	female	4.29569	329.64	11.736	0	5.189	9.219	3.8133	0.0732
**175**	Black / African American	male	1.30048	7.89	0.007	0	0	0	0.0046	0.0137
**176**	Hispanic	female	1.5551	24.14	0.035	0.011	0.022	0.021	0.0274	0.1122
**177**	Black / African American	female	2.51335	67.34	0.009	0	0.025	0.006	0.034	0.0889
**178**	Black / African American	female	1.41547	6.61	0.008	0	0.004	0.007	0.0178	0.032
**179**	Black / African American	male	5.00753	130.84	0.019	0	0.012	0.002	0.0322	0.0285
**180**	Black / African American	male	0.52841	3851.1	0.466	0.3498	1.369	9.946	1.3714	0.2378
**181**	White	male	2.81451	28.86	0.008	0	0.024	0.01	0.0196	0.0911
**182**	Black / African American	female	0.72553	14.54	0	0	0	0	0.0119	0.0389
**183**	Hispanic	female	0.8104	8.5	0	0	0.008	0	0.0248	0.0332
**184**	Black / African American	male	4.89254	19.27	0.014	0	0.013	0.725	0.064	0.0272
**185**	Black / African American	female	4.71732	35.63	0	0	0.009	0.002	0.0215	0.0376
**186**	Black / African American	female	1.10335	1.57	0	0	0	0	0.0108	0.0142
**187**	Black / African American	male	0.52019	4.99	0	0	0.01	0.005	0.0278	0.0374
**188**	Black / African American	female	0.57769	13.24	0	0	0.037	0.001	0.022	0.0334
**189**	Black / African American	male	0.74743	4.41	0	0	0	0	0.003	0.0268
**190**	Black / African American	male	1.01027	7.97	0	0	0	0	0.009	0.0379
**191**	Black / African American	female	3.86585	7.55	0	0	0	0	0.0178	0.0002
**192**	Black / African American	male	5.18275	1324.61	1.742	0	2.237	22.781	2.4582	2.4551
**193**	Black / African American	female	4.47365	13.58	0	0	0	0	0.0051	0.0173
**194**	Hispanic	male	2.05339	5.85	0.281	0.5964	0.046	0.009	0.1765	0.0788
**195**	Hispanic	female	0.75838	22.26	0	0	0	0	0.0091	0
**196**	Black / African American	male	3.68515	89.43	0	0	0	0	0.0132	0.0361
**197**	Black / African American	male	0.62971	7.27	0	0	0	0	0.014	0.0049
**198**	Hispanic	female	0.55305	62.42	0	0	0	0.002	0.0173	0.0322
**199**	Hispanic	female	0.85421	17.85	0.008	0	0.008	0.226	0.1448	0.0213
**200**	Hispanic	female	3.74538	832.2	0.802	0	1.935	32.524	3.3967	0.6315
**201**	Hispanic	female	1.05407	72.52	33.211	0	0.004	0.091	0.0542	0.0712
**202**	Hispanic	male	1.16632	10.06	0.005	0	0.009	0.012	0.0277	0.0184
**203**	Black / African American	female	1.67283	3.9	0.005	0	0.007	0.002	0.0007	0.0367
**204**	Black / African American	male	0.50376	2.39	0	0	0	0	0.0005	0.0399
**205**	Black / African American	male	5.08693	247.58	0.023	0	1.265	0.015	0.0575	0.0711
**206**	Black / African American	male	2.00411	41.79	0.02	0.0068	0.015	0.004	0.0103	0.0882
**207**	Black / African American	male	0.72279	11.42	0.005	0	0.012	0.099	0.0215	0.0126
**208**	Cap Verdean	male	1.02669	28.31	0.007	0	0.01	0.025	0.0268	0.0215
**209**	Caribbean	male	0.59138	1.56	0	0	0	0	0.0177	0.0097
**210**	Black / African American	female	2.67488	4.75	0.011	0	0.004	0.006	0.0098	0.0998
**211**	Hispanic	female	5.01848	20.28	0.007	0	0.011	0.003	0.059	0.0504
**212**	Hispanic	female	0.50103	2.74	0	0	0	0	0.0052	0.0291
**213**	Black / African American	male	4.08761	127.82	0.012	0	0.028	0	0.0221	0.0336

**214**	Black / African American	male	1.32512	20.7	0.006	0	0.013	0.005	0.0091	0.0377
**215**	Hispanic	male	1.00205	5.14	0.009	0	0	0	0.0159	0.0244
**216**	Black / African American	male	1.93018	0.76	0.005	0	0.012	0.001	0.0088	0.1034
**217**	White	female	1.57153	10.59	0.009	0	0.011	0.005	0.0065	0.0611
**218**	White	female	1.23477	6.63	0.008	0	0.006	0	0.0022	0.027
**219**	Black / African American	female	1.51403	36.66	0.024	0	0.029	0.012	0.016	0.0918
**220**	Black / African American	male	0.55305	7.52	0	0	0	0	0.0043	0
**221**	Black / African American	male	0.62697	8.38	0.009	0	0.013	0.008	0.0151	0.1168
**222**	Hispanic	female	0.51198	3.81	0	0	0	0	0.0053	0.0298
**223**	Black / African American	female	4.48734	81.96	0.013	0	0.023	0.003	0.0397	0.0133
**224**	Black / African American	female	5.3744	15.06	0.007	0	0.006	0.001	0.0055	0.0619
**225**	Unknown	male	1.05681	6.15	0.006	0	0.002	0	0.011	0.0284
**226**	Black / African American	male	4.58042	113.82	0.006	0	0.016	0.007	0.0418	0.0364
**227**	Hispanic	male	2.00684	15.56	0.029	0	0.014	0.058	0.0173	0.0874
**228**	Black / African American	male	2.04244	11.86	0.091	0.053	0.04	0.088	0.0971	0.0951
**229**	Unknown	male	0.84326	9.73	0.014	0	0.015	0.002	0.0138	0.0352
**230**	White	female	0.56126	0.03	0	0	0	0	0.0144	0.0133
**231**	Black / African American	female	1.2512	4.54	0.007	0	0.002	0.005	0.0046	0.1118
**232**	Black / African American	male	0.79671	7.27	0.009	0	0.022	0.015	0.0184	0.0499
**233**	Hispanic	female	0.74196	12.32	0.019	0	0.007	0	0.0112	0.0254
**234**	Hispanic	male	0.51198	6.2	0	0	0.036	0	0.0129	0.0049
**235**	Black / African American	male	0.81862	83.23	0.014	0	0.02	0.009	0.0067	0.0328
**236**	Black / African American	male	0.73648	0.01	0	0	0	0	0.0068	0.0262
**237**	Black / African American	male	3.78919	1	0.007	0	0.012	0	0.0056	0.074
**238**	Hispanic	male	0.79945	24.54	0.963	0	0.164	0.602	0.312	0.0278
**239**	Black / African American	male	0.51472	0.85	0	0	0.002	0.001	0.0179	0.0411
**240**	Black / African American	male	1.28679	188.85	0.028	0.0174	0.554	0.033	0.0549	0.0711
**241**	Black / African American	male	4.74743	229.76	0.138	0	0.031	0.016	0.0561	0.0552
**242**	Black / African American	female	0.95277	7.16	0	0	0.009	0.007	0.0167	0.0343
**243**	Black / African American	male	0.51198	10.52	0.011	0	0.002	0.008	0.0095	0.0224
**244**	Black / African American	female	6.12731	44.52	0.026	0.0045	0.022	0.007	0.03	0.0949
**245**	Black / African American	male	0.76112	206.36	0.032	0	0.052	0.009	0.0242	0.0648
**246**	Black / African American	female	0.76112	171.33	0.025	0	0.318	0.014	0.0506	0.0335
**247**	Black / African American	male	3.9206	38.2	0.029	0	0.073	0.103	0.0339	0.0395
**248**	Black / African American	female	4.39425	12.07	0.025	0	0	0	0.0224	0.0498
**249**	White	male	0.78303	5.92	0.01	0	0.004	0.01	0.0118	0.0575
**250**	Hispanic	female	0.65435	8.01	0.011	0	0.005	0.004	0.0035	0.0228
**251**	Black / African American	female	0.53114	7.02	0.006	0	0.008	0	0.0012	0.0343
**252**	Black / African American	female	0.75565	5.53	0.008	0	0.004	0.002	0.0049	0.0649
**253**	White	female	1.60712	8.61	0.28	0.2972	0.046	0.077	0.2116	0.0764
**254**	mixed	female	0.7447	46.35	0.018	0.0047	0.015	0.045	0.0328	0.0984
**255**	Black / African American	female	5.28953	58.17	0.945	10.0486	0.006	0.002	0.0254	0.1283
**256**	Hispanic	male	1.03491	9.89	0.009	0	0.001	0	0.0043	0.1142
**257**	Black / African American	female	5.32786	23.17	0.035	0.095	0	0	0.0367	0.0628

**258**	Hispanic	female	3.77823	1.32	0	0	0	0	0.0023	0.0189
**259**	Black / African American	male	5.37988	16.1	0.007	0	0	0.005	0.0107	0.0809
**260**	Black / African American	male	3.99179	9.32	0	0	0	0	0.0129	0.0439
**261**	Black / African American	female	5.24572	156.41	0.015	0.0022	0.022	0.051	0.0426	0.0991
**262**	Black / African American	female	4.42163	129.73	0.014	0	0.032	0.103	0.052	0.0436
**263**	Hispanic	female	4.02464	48.05	0	0	0	0	0.0081	0.0314
**264**	Black / African American	female	0.75838	13.5	0.004	0.002	0.006	0.004	0.0117	0.0718
**265**	Black / African American	male	4.08761	10.88	0	0	0	0	0.0082	0.0262
**266**	Black / African American	male	0.84873	12.86	0.006	0	0	0	0.0029	0.0723
**267**	Black / African American	female	0.54483	122.88	0.023	0.0016	0.016	0.015	0.0625	0.0702
**268**	Cap Verdean	male	3.51814	44.74	0.005	0	0.003	0.007	0.0045	0.0751
**269**	Hispanic	male	0.73374	39.87	0.01	0.0019	0.01	0.011	0.0909	0.0871
**270**	Hispanic	female	0.76386	1.81	0.006	0	0.004	0.003	0.0162	0.0778
**271**	Black / African American	male	3.74264	2.85	0	0	0	0	0.0078	0.023
**272**	Black / African American	female	0.6653	19.13	0.013	0	0.056	0.002	0.0112	0.0814
**273**	Unknown	male	1.577	0	0	0	0.01	0	0.0076	0.0733
**274**	Black / African American	male	4.81314	464	0.071	0.0311	0.057	0.026	0.17	0.1905
**275**	Hispanic	female	3.24983	11.59	0	0	0	0	0.006	0.0222
**276**	Black / African American	female	0.68172	17.06	0.016	0	0.004	0.001	0.0083	0.0507
**277**	Hispanic	female	0.89802	4.24	0.01	0	0.011	0	0.0104	0.0822
**278**	Black / African American	male	1.06502	9.95	0.001	0	0.012	0	0.0003	0.0609
**279**	Black / African American	female	1.02669	17.25	0.014	0	0.012	0.016	0.0117	0.0964
**280**	Black / African American	female	0.77481	9.7	0.006	0	0.013	0	0.0052	0.08
**281**	Black / African American	female	3.1102	93.91	0	0	0	0	0.0305	0.007
**282**	Black / African American	male	0.82957	8.56	0.009	0	0.015	0	0.0105	0.0982
**283**	Black / African American	female	3.09925	32.21	0	0	0	0	0.0171	0.017
**284**	Black / African American	female	3.71526	0	0	0	0	0	0.0055	0.0458
**285**	Hispanic	female	5.10062	18.5	0.501	0.8117	0.034	0	0.0033	0.0872
**286**	Hispanic	female	0.77207	48.39	0.01	0	0.016	0.036	0.0821	0.0584
**287**	Black / African American	female	0.75017	13.79	0.009	0	0.013	0.002	0.0129	0.092
**288**	Black / African American	male	2.987	8.24	0	0	0	0	0.0113	0
**289**	Black / African American	female	0.97741	3.95	0.014	0	0.023	0	0.0025	0.0968
**290**	Black / African American	male	0.9692	9.91	0.018	0	0.017	0.002	0.0095	0.0907
**291**	Unknown	male	1.09514	42.41	0.041	0.0082	0.446	0.012	0.1712	0.1039
**292**	Black / African American	male	4.01643	145.84	16.553	0	0.016	0.025	0.0918	0.0434
**293**	Black / African American	female	0.77481	12.05	0.021	0	0.02	0.004	0.0158	0.1095
**294**	Hispanic	female	3.49076	94.32	27.57	0	0.015	0	0.0384	0.031
**295**	Hispanic	female	4.68994	267.75	1.311	1.0436	0.027	0.026	0.0537	0.1423
**296**	Black / African American	female	0.74196	43.83	0.077	0.0521	0.057	0.015	0.0445	0.1158
**297**	Cap Verdean	female	2.55441	17.11	0	0	0.023	0.3	0.767	0.0541
**298**	Black / African American	female	0.8104	3.19	0.018	0	0.011	0	0.0147	0.0717
**299**	Black / African American	male	0.95277	2.9	0.002	0	0.02	0	0.014	0.0793
**300**	Black / African American	male	3.00616	85.18	0	0	0.013	0.003	0.0425	0
**301**	Black / African American	male	0.61328	6.55	0.017	0.0027	0.019	0.015	0.0167	0.0819

**302**	Black / African American	male	1.18001	9.4	0.013	0	0.019	0.009	0.0075	0.1123
**303**	Black / African American	male	1.06776	28.68	0.013	0	0.012	0.002	0.024	0.08
**304**	Black / African American	female	2.63381	3.86	0.047	0	0.01	0	0.0415	0.0454
**305**	Black / African American	female	0.82136	23.86	0.011	0	0.02	0.01	0.0559	0.0526
**306**	Black / African American	female	1.01574	5.39	0.011	0	0.022	0.001	0.0106	0.0825
**307**	Black / African American	male	0.74196	2.76	0.009	0	0.011	0.002	0.0081	0.0446
**308**	Caribbean	male	1.013	29.84	0.02	0	0.019	0.015	0.0318	0.0547
**309**	Black / African American	male	3.19233	99.77	0.073	0	0.093	0.006	0.0217	0.0193
**310**	Unknown	male	2.43121	24.7	0	0	0	0.049	0.0242	0.0422
**311**	Hispanic	male	4.43258	3.05	0.008	0	0.021	0	0	0.0758
**312**	Black / African American	female	2.70773	1479.97	0.534	0	1.896	0.136	0.1236	0.0765
**313**	Black / African American	male	0.7885	3.25	0.011	0	0.016	0.002	0.0148	0.0697
**314**	Black / African American	female	3.10198	95.38	0.042	0	0	0.003	0.0208	0.0189
**315**	Black / African American	male	4.5065	2.44	0.006	0	0.014	0	0.0021	0.0831
**316**	Cap Verdean	female	2.55168	18.2	0.044	0	0	0	0.02	0.0294
**317**	Hispanic	male	2.31896	7.83	0.003	0	0.001	0	0.0427	0.0285
**318**	Black / African American	female	0.72827	56.31	0.033	0.021	0.038	0.083	0.0559	0.0631
**319**	Hispanic	female	3.27447	1356.78	108.31	0	4.994	0.464	0.708	0.0735
**320**	Caribbean	female	0.55305	5.06	0.005	0	0.006	0.001	0.0078	0.0941
**321**	Black / African American	female	4.18344	28.78	0.011	0	0.013	0.006	0.0099	0.0749
**322**	Black / African American	male	3.20329	58.53	0.025	0	0.005	0.011	0.0238	0.0325
**323**	Black / African American	female	3.03901	12.73	0	0	0	0	0.0048	0.0043
**324**	Black / African American	female	2.38741	11.79	0.004	0	0.002	0.005	0.0064	0.0241
**325**	Hispanic	female	2.06434	156.19	0.981	0	0.172	0.013	0.0561	0.0253
**326**	Hispanic	female	2.705	5.96	0	0	0	0	0.0482	0.0268
**327**	Hispanic	male	2.43395	11.4	0	0	0	0	0.0106	0.0262
**328**	Black / African American	female	3.48802	201.11	0.038	0	0.034	0.059	0.0665	0.0407
**329**	Hispanic	male	4.16153	41.42	0.227	0.1451	0.074	0.021	0.0693	0.0871
**330**	Black / African American	male	4.65161	4.55	0.018	0.044	0.048	0.004	0.1566	0.0954
**331**	Unknown	male	0.56126	1.67	0.011	0	0.018	0	0.0113	0.0782
**332**	Black / African American	female	3.01164	135.25	0.017	0	0.007	0.022	0.0803	0.024
**333**	Hispanic	male	2.00958	12.15	0	0	0	0	0.0109	0.0253
**334**	Cap Verdean	female	2.10815	26.57	0	0	0	0	0.0083	0.0607
**335**	Black / African American	female	3.15127	416.4	1.333	0	0.179	0.039	0.1318	0.0831
**336**	Hispanic	female	2.65024	38.28	0.008	0	0	0.132	0.2279	0.0318
**337**	White	male	3.88227	0	0.011	0	0	0	0.007	0.0848
**338**	Black / African American	female	2.41752	432.14	2.925	0	0.52	0.034	0.436	0.0596
**339**	Black / African American	male	3.55099	85.72	0.016	0.0093	0.008	0.008	0.0337	0.0927
**340**	Black / African American	female	2.00137	47.31	0.061	0	0	0	0.0148	0
**341**	Black / African American	male	2.36003	26.23	0	0	0.015	0.04	0.0129	0.0113
**342**	Black / African American	male	4.0219	1198.02	4.457	1.8313	101.49	49.27	13.3865	0.5908
**343**	Hispanic	female	4.02738	24.78	5.096	8.2311	0.019	0.015	0.0214	0.0734
**344**	Black / African American	male	3.44695	21.22	0.006	0	0.005	0	0.0055	0.087
**345**	Black / African American	female	2.423	76.92	0.446	0	0.038	0	0.0224	0.0351

**346**	Black / African American	female	2.01506	3.14	0.941	0	0.126	0.008	0.339	0.0843
**347**	Black / African American	male	4.03285	35.98	0.022	0	0.014	0.011	0.0153	0.1047
**348**	Black / African American	male	2.78166	47.39	0	0	0	0.103	0.0181	0.0559
**349**	Black / African American	male	3.07461	12.23	0.13	0	0.049	0.001	0.01	0.0241
**350**	Black / African American	female	4.24093	14.41	0.01	0	0.008	0.005	0.0074	0.0709
**351**	White	female	3.04997	86.66	0.023	0	0.016	0.029	0.031	0.0556
**352**	Black / African American	female	2.57084	11.8	0	0	0	0.017	0.016	0.0204
**353**	Black / African American	female	2.84189	160.64	0.766	0	0.37	0.646	0.8717	0.092
**354**	Black / African American	female	3.01711	21.06	0.015	0	0.005	0.005	0.0067	0.0386
**355**	Black / African American	female	1.76318	26.15	0	0	0	0.019	0.0297	0.0266
**356**	Black / African American	female	3.21424	44.38	0.001	0	0.004	0.032	0.0153	0.0379
**357**	Black / African American	male	4.02464	37.27	0.013	0	0.007	0.431	0.042	0.1473
**358**	Black / African American	male	2.51608	10.34	0.197	0	0	0	0.0088	0
**359**	Black / African American	female	1.90281	71.63	0.045	0	0	0	0.0205	0.0314
**360**	Black / African American	male	1.74949	273.18	0.018	0	0.211	0.046	0.1137	0
**361**	Unknown	female	2.16564	23.94	0	0	0.015	0	0.0128	0.0122
**362**	Black / African American	male	3.8987	4.89	0.015	0	0.009	0.004	0.0058	0.1041
**363**	Black / African American	female	3.68241	0	0.034	0.0095	0.012	0.003	0.0221	0.1464
**364**	Cap Verdean	female	3.62765	173.41	8.614	10.2382	0.036	0.063	0.0304	0.1373
**365**	Black / African American	female	2.57084	20.39	0.003	0	0.002	0	0.0155	0.0135
**366**	Black / African American	female	4.29569	0	0	0	0	0	0.0061	0.0605
**367**	Hispanic	female	2.17659	145.8	0.012	0	0.004	0	0.0321	0.0475
**368**	Hispanic	male	1.99042	0.43	0	0	0	0	0.0113	0.0285
**369**	Hispanic	female	1.54962	8.75	0	0	0	0.002	0.0249	0.0379
**370**	Black / African American	male	3.39767	125.87	1.25	0.6599	0.164	0.02	0.0253	0.0939
**371**	Black / African American	male	4.19713	128.43	0.016	0	0.013	0.007	0.0288	0.0859
**372**	Black / African American	female	3.99452	18.38	0.012	0	0.016	0.006	0.0145	0.09
**373**	Hispanic	female	1.50582	20.97	0	0	0.009	0.01	0.0361	0.0355
**374**	Hispanic	female	1.45106	1224.3	0.161	0	2.728	0.396	0.5771	0.0677
**375**	Black / African American	male	1.43463	3.01	0	0	0	0	0.0179	0.01
**376**	Black / African American	female	3.19781	66.29	0.02	0.0014	0.006	0.004	0.0068	0.1222
**377**	Ascian/ Pacific	male	1.52498	5.26	0	0	0	0	0.0103	0.0283
**378**	Hispanic	male	3.26078	916.75	0.212	0.4161	1.676	39.432	3.9975	0.1614
**379**	Black / African American	male	1.50582	28.38	0	0	0	0	0.0249	0.016
**380**	Black / African American	male	3.14305	1.01	0.001	0	0	0	0.0032	0.0917
**381**	Black / African American	male	1.82341	5.69	0	0	0	0	0.0049	0.01
**382**	Black / African American	male	3.49624	24.42	0.175	0.0926	0.024	0	0.0094	0.1326
**383**	Black / African American	female	3.43326	110.45	6.67	20.7629	0.573	0.073	0.0769	0.1235
**384**	Black / African American	male	2.00411	271.4	0.012	0	0.024	0.058	0.0538	0.0083
**385**	Cap Verdean	female	1.69747	19.17	0.076	0	0	0	0.0141	0.0367
**386**	Ascian/ Pacific	male	2.00958	12.66	0	0	0	0	0.0056	0.0044
**387**	Hispanic	male	1.29227	13.17	0	0	0	0	0.001	0.0597
**388**	Hispanic	male	1.71663	13.57	0.359	0	0.074	0	0.0991	0.0292
**389**	Hispanic	male	1.23751	7.58	0.049	0	0.029	0	0.0557	0.0256

**390**	Hispanic	male	3.11567	10.85	0.026	0.0041	0.017	0.011	0.0126	0.0965
**391**	Hispanic	male	1.79877	13.14	0.006	0	0.02	0	0.0687	0.0094
**392**	Black / African American	male	1.54689	10.29	0.001	0	0	0	0.0083	0.0287
**393**	Black / African American	female	1.13895	7.05	0	0	0	0	0.0103	0.066
**394**	Hispanic	female	1.0924	18.02	0.019	0	0.01	0	0.0082	0.029
**395**	Black / African American	female	1.33607	110.88	0	0	0	0	0.0176	0.0521
**396**	Black / African American	male	3.02259	14.8	0.025	0.0133	0.032	0.015	0.0238	0.1234
**397**	Black / African American	male	1.59343	176.12	1.005	0	1.507	2.516	0.1652	0.3643
**398**	Black / African American	male	1.99316	195.47	0.653	0	0.229	0.024	0.0669	0.0296
**399**	Black / African American	female	0.97194	80.96	0	0	0.011	0	0.021	0.0186
**400**	Black / African American	female	2.20945	232.8	0.021	0	0.039	0.027	0.137	0.0418
**401**	White	female	1.08145	27.94	0	0	0.001	0	0.017	0.0219
**402**	Cap Verdean	female	1.81793	24.79	0	0	0	0	0.0056	0.0176
**403**	Black / African American	female	3.01711	1280.08	2.429	1.6734	7.718	0.112	1.0838	0.1533
**404**	Black / African American	male	1.00205	20.54	0	0	0	0	0.015	0
**405**	Hispanic	female	1.1937	493.78	0.049	0	0.239	1.172	1.2583	0.0678
**406**	Black / African American	female	1.15537	475.66	0.096	0	0.252	0.093	0.2556	0.0907
**407**	Black / African American	male	1.86995	57.23	0.011	0	0.011	0.003	0.0212	0.0543
**408**	Black / African American	male	2.30253	285.75	0.018	0.0128	0.086	0.008	0.0172	0.088
**409**	Black / African American	female	1.50034	17.88	0	0	0	0	0.0123	0.0076
**410**	White	male	2.00411	7.29	0.006	0	0.013	0.008	0.0075	0.0342
**411**	Black / African American	male	0.91718	6.56	0.006	0	0.017	0	0.0223	0.0817
**412**	Hispanic	female	1.74127	43.41	0.009	0	0.025	0.006	0.0107	0.0199
**413**	Cap Verdean	male	1.21013	16.07	0.003	0	0	0	0.014	0.0435
**414**	Hispanic	male	2.77344	0.32	0.035	0.0091	0.01	0	0.1228	0.1318
**415**	White	male	0.56674	33.3	0.009	0	0.005	0.017	0.0365	0.0266
**416**	Unknown	female	1.03765	2.36	0	0	0	0	0.0045	0.0074
**417**	Black / African American	female	2.00137	1.36	0.008	0	0	0.004	0.0051	0.062
**418**	Black / African American	female	1.27036	137.39	0.003	0	0.194	0.054	0.1652	0.0143
**419**	Ascian/ Pacific	female	1.47296	12.53	0	0	0	0	0.0055	0.0222
**420**	White	female	0.82409	6.91	0	0	0	0	0.0145	0.0265
**421**	Black / African American	female	2.07529	0	0	0	0	0	0.0045	0.007
**422**	Caribbean	female	0.61054	0.08	0	0	0	0	0.0041	0.0261
**423**	Black / African American	female	1.56605	15.38	0.007	0	0.015	0.009	0.0201	0.0364
**424**	Black / African American	male	2.13279	10.42	0.01	0	0.016	0	0.0219	0.0708
**425**	Black / African American	female	1.10335	16.88	0.006	0	0.005	0	0.0083	0.007
**426**	Hispanic	female	1.56879	5.42	0.005	0	0.01	0	0.0056	0.0369
**427**	Hispanic	female	0.74743	68.23	0.028	0.0093	0.055	0.011	0.0369	0.1079
**428**	Hispanic	male	1.05955	4.57	0	0	0	0	0.0069	0
**429**	Black / African American	female	0.85695	0	0	0	0	0	0.0044	0.0234
**430**	Black / African American	male	0.82136	57.75	0.013	0.0028	0.145	0.012	0.0214	0.0967
**431**	Black / African American	female	2.75975	12.6	0.011	0	0.01	0.004	0.0067	0.0973
**432**	Black / African American	female	1.54689	140.94	0.014	0	0.015	0.006	0.0179	0.0312
**433**	Black / African American	female	0.52293	9.19	0	0	0	0.004	0.0039	0.0487

**434**	Hispanic	male	1.60438	7.73	0.011	0	0.009	0	0.0103	0.0333
**435**	Black / African American	female	1.74127	89.15	0.547	0	0.027	0.02	0.0274	0.0589
**436**	Black / African American	female	2.29979	24.16	0.009	0	0.003	0.008	0.0098	0.0575
**437**	Black / African American	female	1.88912	25.92	0.009	0	0	0	0.0005	0.0523
**438**	Black / African American	male	1.25667	52.47	0	0	0	0	0.0159	0.0118
**439**	White	female	0.61875	1.97	0	0	0.005	0	0.0095	0.0151
**440**	Black / African American	female	0.99932	51.9	0	0	0.006	0	0.0162	0
**441**	Black / African American	male	0.50103	5.48	0	0	0	0	0.0185	0.0338
**442**	Black / African American	female	0.74196	1391.7	0.159	0	0.468	0.173	0.2261	0.0959
**443**	Hispanic	female	0.5859	4.55	0	0	0	0	0	0.0219
**444**	Black / African American	female	0.77481	12.94	0.03	0.0137	0.035	0.032	0.0299	0.0893
**445**	Hispanic	female	1.01027	31.12	0	0	0	0	0.0086	0.0282
**446**	Cap Verdean	female	0.73101	8.39	0.019	0.0112	0.015	0.003	0.0107	0.1043
**447**	Black / African American	male	0.55852	3.45	0	0	0	0	0.0058	0.0447
**448**	Black / African American	male	1.1718	385.95	0.021	0	0.023	0.013	0.0332	0.0213
**449**	Black / African American	male	1.00479	46.86	0	0	0.006	0	0.0066	0.0044
**450**	Black / African American	male	0.75565	71.32	0	0	0	0	0.0457	0.0046
**451**	Hispanic	female	0.77755	5.07	0.001	0	0	0	0.0103	0.0339
**452**	Hispanic	male	1.61807	28.15	0.018	0	0.014	0.006	0.0138	0.032
**453**	Black / African American	male	1.34155	280.93	0.035	0	0.034	0.034	0.0545	0.032
**454**	White	female	0.55578	19.28	0	0	0.095	0	0.3801	0.0256
**455**	White	female	1.68378	14.51	0.11	0.1658	0.003	0.008	0.0047	0.0663
**456**	Black / African American	male	1.53593	11.97	0.007	0	0.014	0.014	0.0097	0.021
**457**	Black / African American	male	0.84326	80.26	0.007	0	0.009	0.002	0.0142	0.0313
**458**	Black / African American	female	1.00479	110.81	0	0	0.121	0.231	0.074	0.0201
**459**	Black / African American	male	1.06776	40.31	0	0	0.027	0	0.0065	0.0401
**460**	Black / African American	female	0.75291	4.02	0	0	0	0	0.0056	0.0317
**461**	Hispanic	female	0.74196	1.35	0	0	0	0	0.0045	0.0224
**462**	White	male	0.85695	0	0.016	0	0	0	0.0073	0.0852
**463**	Hispanic	female	0.75838	21.03	0	0	0	0	0.0289	0
**464**	Black / African American	female	0.59138	16.14	0	0	0	0	0.0103	0
**465**	White	female	0.78303	2.8	0	0	0	0	0.0026	0.0111
**466**	Black / African American	male	1.20465	34.99	0.008	0	0.014	0.003	0.0154	0.0323
**467**	Black / African American	female	0.55305	245.15	0.709	0	0.098	20.365	0.6284	0.0389
**468**	Black / African American	female	1.02669	9.07	0.019	0	0.018	0.002	0.0044	0.0197
**469**	Black / African American	female	1.13895	17.97	0.006	0	0.008	0.009	0.0123	0.0202
**470**	Black / African American	male	1.36619	7.74	0.009	0	0.007	0.001	0.0087	0.0231
**471**	Black / African American	male	1.13895	47.94	0.005	0	0.005	0.007	0.0239	0.0374
**472**	Black / African American	female	1.2512	4.59	0.007	0	0.006	0	0.0075	0.0278
**473**	Cap Verdean	male	0.83504	65.5	0	0	0	0	0.0154	0.0224
**474**	Black / African American	female	1.48939	139.82	0.034	0.0318	0.086	0.018	0.0509	0.0741
**475**	Unknown	male	0.76934	14.78	0.016	0	0.028	0.012	0.0279	0.0196
**476**	Black / African American	female	2.07803	29.64	0.021	0	0.069	0.007	0.3109	0.125
**477**	Black / African American	female	1.76044	13.62	0	0	0	0.063	0.0084	0.0386

**478**	Black / African American	male	0.75291	2.49	0.006	0	0	0.002	0.002	0.0823
**479**	Black / African American	male	0.55031	24.63	0	0	0	0	0.01	0.0174
**480**	Black / African American	female	1.88638	15.75	0.001	0	0.007	0.109	0.1096	0.1126
**481**	Black / African American	male	1.50308	0.03	0	0	0.001	0	0	0.0644
**482**	Black / African American	male	0.64613	4.05	0.004	0	0.006	0	0.0148	0.0188
**483**	Black / African American	female	1.54415	361.98	0.632	0.6816	1.615	59.464	5.9	0.0747
**484**	Black / African American	male	1.44011	85.77	0.02	0.0083	0.124	0.023	0.0759	0.0533
**485**	Black / African American	female	0.71732	4.76	0	0	0	0	0.0076	0.0277
**486**	Black / African American	male	0.80219	35.68	0	0	0.016	0	0.0223	0.0221
**487**	Hispanic	male	0.52019	2.22	0	0	0	0	0.009	0.0004
**488**	Black / African American	female	0.564	4.55	0	0	0	0	0.0051	0.0483
**489**	Black / African American	female	0.52567	14.33	0	0	0	0	0.008	0.0336
**490**	Black / African American	male	1.013	0.82	0.017	0	0.005	0.001	0.0326	0.049
**491**	Black / African American	male	0.87337	0.38	0.011	0	0	0	0.0269	0.0254
**492**	Black / African American	female	0.73922	9.34	0.003	0	0.009	0	0.0164	0.0312
**493**	Black / African American	female	0.54757	3.46	0	0	0	0	0.012	0.0199
**494**	White	female	0.62971	23.22	0.028	0	0.002	0.388	0.0396	0.0474
**495**	Black / African American	male	0.75838	30.78	0.028	0	0.009	0.024	0.1105	0.0265
**496**	Black / African American	male	0.89254	4.17	0.02	0	0.007	0.001	0.005	0.036
**497**	Hispanic	female	0.87337	130.47	0.03	0	0.138	0.089	0.0652	0.0801
**498**	Black / African American	male	0.846	2.84	0.007	0	0.005	0	0.0057	0.0227
**499**	Hispanic	male	1.05681	20.83	0.007	0	0.006	0.005	0.0227	0.0352
**500**	Black / African American	male	0.76386	34.46	0.01	0	0.017	0.001	0.0143	0.0346
**501**	Black / African American	male	1.22656	3.41	0.01	0	0.002	0	0.0033	0.0696
**502**	Black / African American	female	0.85695	112.59	0.016	0	0.016	0.003	0.017	0.0572
**503**	Black / African American	male	0.7447	61.93	0.008	0	0.122	0.008	0.017	0.0269
**504**	Hispanic	female	0.77207	319.31	0.023	0.0187	0.061	0.032	0.1329	0.1092
**505**	Black / African American	female	1.25941	0.74	0.008	0	0.005	0	0.0069	0.0759
**506**	Black / African American	female	0.69815	65.26	0.003	0	0.022	0.005	0.0235	0.0239
**507**	Black / African American	male	0.5065	125.01	0.012	0	0.025	0.016	0.0251	0.0316
**508**	Hispanic	male	0.78576	15.2	0.221	0	0.028	0.003	0.0862	0.0316
**509**	Hispanic	male	1.2512	7.38	0.001	0	0.001	0.003	0.0065	0.07
**510**	White	male	0.71458	3.84	0.004	0	0.005	0	0.0015	0.0294
**511**	Black / African American	female	1.13073	261.23	0.028	0.0197	0.206	0.058	0.1301	0.1463
**512**	Black / African American	female	1.24298	10.57	0.005	0	0.089	0.003	0.0124	0.0753
**513**	Black / African American	male	1.02943	26.53	0	0	0.002	0.001	0.0031	0.0795
**514**	Ascian/ Pacific	female	0.83504	17.92	0.003	0	0.008	0.002	0.0114	0.0316
**515**	Hispanic	male	1.32238	11.17	0.013	0	0.005	0	0.0005	0.1066
**516**	Black / African American	female	0.6872	9.2	0.01	0	0.002	0.025	0.0512	0.0255
**517**	Cap Verdean	male	1.68104	3.04	0.026	0.0125	0.001	0	0.0358	0.1232
**518**	Black / African American	male	0.7666	73.86	0.011	0	0.082	0	0.019	0.0301
**519**	Hispanic	female	0.75565	37.96	0.013	0	0.018	0.007	0.0184	0.0328
**520**	Black / African American	female	0.50376	5.43	0.007	0	0.016	0.004	0.0143	0.013
**521**	Black / African American	male	0.95551	25.27	0.007	0	0.036	0.002	0.0175	0.0648

**522**	Black / African American	female	1.51951	29.81	0.031	0.0049	0.09	0.043	1.8659	1.4507
**523**	Black / African American	female	0.75017	5.06	0.016	0	0.012	0.004	0.004	0.0343
**524**	Black / African American	female	1.68378	41.89	0.034	0	0.013	0.005	0.1083	0.057
**525**	Cap Verdean	male	1.51951	90.6	0.078	0.0338	0.14	0.017	0.0332	0.0934
**526**	Hispanic	male	0.53388	1.47	0.008	0	0.013	0	0.0036	0.0319
**527**	Black / African American	female	1.04038	7.99	0	0	0	0.004	0.0054	0.077
**528**	Unknown	female	0.58316	13.9	0.006	0	0.007	0.003	0.0116	0.0326
**529**	Black / African American	male	0.50103	0.7	0.004	0	0.005	0	0.0015	0.0537
**530**	Black / African American	female	0.76112	13.31	0.008	0	0.015	0	0.0058	0.0422
**531**	Black / African American	male	0.5065	3.1	0.006	0	0.007	0	0.0068	0.0219
**532**	Black / African American	male	1.85079	1.33	0.003	0	0.014	0.002	0.0178	0.0571
**533**	Ascian/ Pacific	female	0.52841	7.91	0.011	0	0.005	0.004	0.0102	0.0259
**534**	Unknown	female	0.75838	15.85	0.006	0	0.066	0.037	0.0187	0.0666
**535**	Black / African American	male	0.5859	0.25	0.001	0	0.002	0	0.0061	0.0365
**536**	Black / African American	male	1.03491	127.41	0.131	0.0902	0.035	0.026	0.0478	0.0707
**537**	Black / African American	female	0.73374	25.96	0.003	0	0.032	0.004	0.003	0.085
**538**	Hispanic	male	1.00205	14.85	0.015	0	0.008	0.002	0.0151	0.0629
**539**	Black / African American	male	1.22108	3.33	0.011	0	0.018	0.006	0.0225	0.1075
**540**	Hispanic	male	0.73374	25.31	0.006	0	0.019	0.001	0.0044	0.0584
**541**	Black / African American	female	1.74675	10.04	0.011	0	0.017	0.012	0.0224	0.0513
**542**	Black / African American	female	0.80493	41.63	0.01	0.0006	0.027	0.021	0.0207	0.0931
**543**	Unknown	female	1.10883	44.54	0.018	0	0.037	0.022	0.0841	0.0877
**544**	Hispanic	male	0.46543	10.59	0.008	0	0.005	0.002	0.0078	0.0354
**545**	Unknown	male	0.80493	26.5	0.008	0.0015	0.005	0.008	0.0198	0.1023
**546**	Hispanic	female	1.01574	18.61	0.006	0	0.004	0.007	0.0107	0.073
**547**	White	male	0.59138	1.72	0.017	0	0.008	0	0.0073	0.0419
**548**	Black / African American	female	1.42916	23.71	0.003	0	0.048	0	0.0129	0.0701
**549**	Black / African American	male	0.9911	3.49	0.01	0	0	0	0.0032	0.0859
**550**	Ascian/ Pacific	male	0.54483	48.16	0.004	0	0.028	0.002	0.0108	0.0278
**551**	Black / African American	male	1.04038	5.3	0.011	0	0	0.003	0.0142	0.1122
**552**	Hispanic	male	1.03765	28.01	0.018	0.0077	0.021	0.008	0.0343	0.0877
**553**	Black / African American	male	0.93087	0.68	0.015	0	0.001	0	0.0014	0.076
**554**	Black / African American	male	0.82409	3.03	0.023	0.0198	0.016	0.012	0.0364	0.0802
**555**	Black / African American	female	1.58795	63.82	0.009	0	0.03	0.007	0.0383	0.053
**556**	Black / African American	female	1.01848	78.54	0.011	0.0021	0.051	0.013	0.038	0.1009
**557**	Caribbean	male	0.76386	9.75	0.002	0	0.004	0.001	0.0126	0.0696
**558**	Black / African American	female	1.31964	8.13	0.048	0.0422	0.016	0.001	0.0911	0.067
**559**	Black / African American	female	0.78303	43.09	0.013	0	0.008	0.008	0.0275	0.1116
**560**	Hispanic	female	1.01848	16.27	0.017	0.0068	0.022	0.017	0.0505	0.059
**561**	Black / African American	female	0.57769	2.18	0.018	0.007	0.008	0.01	0.0183	0.0685
**562**	Black / African American	male	0.79945	4.28	0.006	0	0.006	0	0.0008	0.0749
**563**	Black / African American	male	0.83231	2.98	0.014	0	0.008	0	0.0075	0.1028
**564**	Black / African American	female	1.01848	47.84	0.06	0.0647	0.023	0.017	0.3284	0.1182
**565**	White	male	0.97741	5.47	0.018	0	0.016	0.003	0.0101	0.0979

**566**	Black / African American	female	0.66804	41.46	0.035	0.011	0.023	0.051	0.0448	0.0609
**567**	Hispanic	female	0.65708	622	0.449	0.3516	0.675	48.014	1.865	0.0787
**568**	Black / African American	male	0.86242	94.11	0.017	0.0045	0.133	0.015	0.0832	0.0839
**569**	Black / African American	male	1.38261	7.51	0.01	0	0.013	0	0.011	0.0683
**570**	Black / African American	male	0.74196	7.59	0.013	0	0.002	0.014	0.0137	0.0942
**571**	Cap Verdean	male	0.72005	15.64	0.01	0	0.004	0.003	0.005	0.0736
**572**	Cap Verdean	male	0.79398	6.55	0.01	0	0.007	0.002	0.0055	0.0993
**573**	Black / African American	female	0.98563	79.33	0.069	0.0582	0.064	0.079	0.1642	0.1036
**574**	Black / African American	female	0.51745	5.85	0.007	0	0.006	0.001	0.0158	0.08
**575**	Black / African American	male	1.4976	3.36	0.001	0	0.005	0	0.01	0.0758
**576**	White	male	0.75565	7.19	0.007	0	0.006	0	0.0032	0.1095
**577**	Hispanic	female	1.10883	21.13	0.028	0	0.028	0.009	0.0134	0.0749
**578**	Hispanic	female	1.01574	201.69	5.645	2.8827	1.317	0.629	0.9374	1.3748
**579**	Hispanic	male	0.76112	1.38	0.004	0	0.008	0.003	0.0114	0.0855
**580**	Black / African American	male	0.8679	127.66	0.033	0.0131	0.047	0.014	0.0398	0.1378
**581**	Black / African American	female	0.75291	50.13	0.012	0.0035	0.019	0.011	0.0328	0.0567
**582**	Black / African American	female	0.87337	4.76	0.022	0.0093	0.021	0.013	0.2345	0.1166
**583**	Unknown	female	0.53388	48.04	0.017	0.0003	0.025	0.026	0.1769	0.0705
**584**	Unknown	male	0.73374	7.82	0.007	0	0.013	0.002	0.0094	0.1156
**585**	Cap Verdean	male	0.72827	7.3	0.006	0	0.008	0.002	0.0398	0.0644
**586**	Black / African American	male	0.82957	5.96	0.024	0	0.022	0.008	0.0143	0.1468
**587**	Hispanic	male	0.7447	1.52	0.013	0	0.004	0.003	0.0086	0.0916
**588**	Hispanic	female	0.5065	71.26	0.021	0	0.004	0.004	0.0212	0.0532
**589**	Black / African American	female	0.75017	2.93	0.018	0.0024	0.012	0.006	0.008	0.1296
**590**	Black / African American	male	0.50103	10.64	0.006	0	0.009	0.001	0.0469	0.0932
**591**	Unknown	female	0.76934	12.85	0.025	0	0.014	0.007	0.0232	0.1323
**592**	Black / African American	male	0.62149	9.66	0.01	0	0.014	0.004	0.0317	0.0756
**593**	Cap Verdean	male	0.75017	3.36	0.023	0	0.013	0.005	0.0108	0.0821
**594**	Hispanic	female	0.82957	10.15	0.007	0.192	0.007	0.682	0.093	0.0664
**595**	Unknown	female	0.78576	11.35	0.012	0	0.02	0.005	0.0133	0.0744
**596**	Cap Verdean	male	0.54209	11.91	0.013	0	0.01	0	0.0046	0.1199
**597**	Hispanic	female	0.98015	106.92	0.019	0	0.03	0.01	0.0384	0.0911
**598**	Black / African American	male	1.25941	539.81	0.061	0.0355	1.474	0.075	0.1374	0.1908
**599**	White	male	0.7091	47.34	0.028	0.0003	0.017	0.014	0.0266	0.0886
**600**	Hispanic	female	1.0705	66.68	0.034	0.0191	0.083	0.048	0.0642	0.0941
**601**	Black / African American	female	0.76386	9.05	0.022	0.157	0.035	0.013	0.0334	0.0829
**602**	Black / African American	male	0.95825	190.23	0.04	0.0036	0.138	0.012	0.0852	0.038
**603**	Black / African American	male	0.74743	15.32	0.035	0.1196	0.035	0.016	0.0359	0.0987
**604**	Black / African American	female	0.83504	51.74	0.019	0	0.018	0.007	0.0204	0.1102
**605**	Black / African American	female	1.12252	14.71	6.998	3.6258	0.016	0.017	0.0283	0.0653
**606**	Black / African American	male	0.78029	3.85	0.011	0	0.001	0	0.013	0.1357
**607**	Black / African American	male	1.05407	300.88	0.042	0.015	0.757	29.541	9.5016	0.0622
**608**	Hispanic	female	0.86516	14.62	0	0	0	0	0	0.0595
**609**	Black / African American	male	1.08693	1.63	0.005	0	0.013	0	0.0087	0.0591

**610**	Hispanic	female	0.78029	9.84	0.019	0	0.016	0.005	0.0074	0.0841
**611**	Black / African American	female	1.00479	7.04	0	0	0.01	0.002	0.016	0.0527
**612**	Black / African American	male	0.73648	5.2	0.017	0.0006	0.012	0.006	0.0198	0.0733
**613**	Cap Verdean	male	1.01574	3.69	0	0	0.011	0.003	0.0094	0.1134
**614**	Hispanic	male	0.51472	31.57	0.011	0	0.012	0.002	0.0158	0.1173
**615**	Black / African American	male	0.92813	3.42	0.019	0	0.018	0	0.0067	0.0839
**616**	White	female	0.80767	1.69	0.005	0	0.009	0	0.0024	0.0629
**617**	Hispanic	male	0.75017	2.84	0.008	0	0.013	0	0.0033	0.0761
**618**	Black / African American	female	0.97467	1.7	0.006	0	0.008	0.002	0.0145	0.0697
**619**	Hispanic	male	1.04038	7.59	0.005	0	0.011	0	0.017	0.076
**620**	Black / African American	female	0.55031	0	0.011	0	0.009	0	0.0124	0.1619
**621**	Black / African American	male	0.87885	3.5	0.013	0	0.012	0.002	0.0133	0.0845
**622**	Black / African American	male	0.62971	9.84	0.007	0	0.012	0.002	0.0033	0.0781
**623**	Cap Verdean	female	0.89254	30.5	0.012	0	0.013	0.005	0.0163	0.0791
**624**	Black / African American	female	0.50103	25.66	0.012	0.0023	0.035	0.005	0.0147	0.0691
**625**	Cap Verdean	male	0.75017	14.94	0.002	0	0.022	0	0.0269	0.0759
**626**	Cap Verdean	female	0.75565	3.62	0.007	0	0.011	0	0.0037	0.1005
**627**	Cap Verdean	male	0.64887	22.44	0.01	0	0.031	0.018	0.0433	0.0779
**628**	Hispanic	female	0.75291	11.39	0.012	0	0.014	0.001	0.008	0.0855
**629**	Hispanic	male	0.79945	0.61	0.004	0	0.006	0	0.0106	0.076
**630**	Unknown	female	0.77207	7.67	0.014	0	0.011	0.005	0.0293	0.0783
**631**	Black / African American	female	0.69268	114.68	0.017	0	0.051	0.009	0.1782	0.0896

Table S3. Children with one or multiple (2-6) allergens

No. of positive to tested allergens	No. of Subjects (207)	% of total population (100%)
**1**	100	48.3
**2**	47	22.7
**3**	25	12.1
**4**	17	8.2
**5**	10	4.8
**6**	8	3.9
